# The ethical challenges in the integration of artificial intelligence and large language models in medical education: A scoping review

**DOI:** 10.1371/journal.pone.0333411

**Published:** 2025-10-22

**Authors:** Xinrui Li, Xiaodan Yan, Han Lai

**Affiliations:** 1 Hospital of Chengdu University of Traditional Chinese Medicine, Chengdu, China; 2 Chengdu University of Traditional Chinese Medicine, Chengdu, China; Helwan University Faculty of Education, EGYPT

## Abstract

With the rapid development of artificial intelligence (AI), large language models (LLMs), such as ChatGPT have shown potential in medical education, offering personalized learning experiences. However, this integration raises ethical concerns, including privacy, autonomy, and transparency. This study employed a scoping review methodology, systematically searching relevant literature published between January 2010 and August 31, 2024, across three major databases: PubMed, Embase, and Web of Science. Through rigorous screening, 50 articles which met inclusion criteria were ultimately selected from an initial pool of 1,192 records. During data processing, the Kimi AI tool was utilized to facilitate preliminary literature screening, extraction of key information, and construction of content frameworks. Data reliability was ensured through a stringent cross-verification process whereby two independent researchers validated all AI-generated content against original source materials. The study delineates ethical challenges and opportunities arising from the integration of AI and LLMs into medical education, identifying seven core ethical dimensions: privacy and data security, algorithmic bias, accountability attribution, fairness assurance, technological reliability, application dependency, and patient autonomy. Corresponding mitigation strategies were formulated for each challenge. Future research should prioritize establishing dedicated ethical frameworks and application guidelines for AI in medical education while maintaining sustained attention to the long-term ethical implications of these technologies in healthcare domains.

## Introduction

Artificial intelligence (AI) originally referred to as the simulation of human intelligence processes through algorithms and data analysis originated in the 1950s [1]. The emergence of ChatGPT in 2022 catalyzed the rapid development of large AI-based language models (LLMs), which shows AI’s rapid growth and progress [2]. AI and LLMs show promising applications in various fields, such as scientific research, statistical analysis, and machine translation, especially in health care and medical education [3–5].

With their powerful data processing and natural language understanding capabilities, AI and LLMs are changing how medical knowledge is acquired, taught, and applied [6].This transformation aligns closely with the core objectives of medical education, which aims not only to impart knowledge but also to cultivate clinical skills, professional attitudes, and ethical decision-making abilities—reflecting its unique focus on shaping medical professional values [7]. A complex and extensive body of knowledge is required for medical students and staff, and AI and LLMs can achieve this goal by integrating and analyzing vast datasets [8]. AI leverages machine learning and computer vision to focus on medical operational skill training in medical education, such as virtual surgical simulation, whereas LLMs utilize their language comprehension and generation capabilities to emphasize knowledge integration and interactive reasoning, primarily driving clinical knowledge synthesis and decision-making exercises, such as the simulation of complex physician-patient communication scenarios through natural language interactions. Studies have shown that AI and LLMs have the potential to enhance the learning experience and quality of education for medical students [9,10].

However, integrating AI and LLMs into medical education also faces ethical challenges [11], which should be contextualized within the established framework of medical ethics principles—namely respect for autonomy, beneficence, non-maleficence, and justice [12]. Patients’ right to know and privacy protection, the accessibility and affordability of technology, and the definition of responsibility in the context of AI-assisted medical education are critical ethical issues that need to be addressed urgently [13–15]. As we stand on the cusp of a new era of medical education, we must address these challenges with foresight and prudence.

## Objectives

In this study, we conducted a scoping review to explore the ethical challenges faced in integrating AI and LLMs in medical education. We set two objectives: first, to synthesize existing literature elucidating critical ethical challenges arising from AI and LLM applications in medical education; second, to explore solutions, outline actionable implementation pathways, and establish a foundational discourse for responsibly leveraging these technologies to support evidence-based decision-making and educational practices.By achieving these objectives, we aimed to advance the responsible and effective integration of AI and LLMs into medical education..

## Methods

### Study design and registration

This scoping review was conducted according to the framework established by Arksey and O’Malley (Arksey et al., 2005) as well as the PRISMA Extension for Scoping Reviews (PRISMA-ScR) [16]. The PRISMA-ScR checklist is shown in S1 File. The study has been pre-registered on the OSF platform (https://doi.org/10.17605/OSF.IO/65TYD).

### Search strategy

The researchers systematically searched relevant articles published from 2010 to August 31, 2024 in the PubMed, Embase, and Web of Science databases, with the year 2010 chosen as the starting point to capture breakthrough advancements in deep learning, the rise of structured electronic health data, and the transition of AI applications in healthcare from theoretical frameworks to clinical validation. We used a combination of subject terms and free text terms, and adjusted the search according to the characteristics of each database. The search terms used are listed in Table 1. The complete search strategy can be found in S2 File.

**Table 1:** The terms used in the database search.

	Search term
AND	Medical OR Medicine
	Education OR Educate
AND	Artificial Intelligence OR AI OR Machine Learning OR ML OR Deep Learning OR Data Analytics OR Natural Language Processing OR Neural Networks OR Pattern Recognition OR Data Mining OR Computer Vision OR Reinforcement Learning OR Automated Reasoning OR Cognitive Computing OR Machine Intelligence OR Intelligent Systems OR Intelligent Control OR Large language Model OR LLM OR Natural Language model OR NLM OR Chatgpt OR multimodal OR multimodality

### Inclusion and exclusion criteria

#### Inclusion criteria.

(1) The article discusses the application of the integration of AI and LLMs in medical education, and explores ethical issues in the main text. Studies on non-education-related AI technologies (e.g., genomic bioinformatics analysis) are excluded. (2) The article explicitly mention or implicitly address ethical challenges related to AI implementation (e.g., data privacy, algorithmic bias, accountability disputes), with substantial discussion beyond superficial keyword references. (3) The article was published from January 1, 2010 to August 31, 2024.

#### Exclusion criteria.

(1) To prioritize the unique demands of physician education,articles that solely focus on non-clinical medical education (e.g., nursing, pharmacy, dentistry) without addressing clinical medical education are excluded;(2) Articles that mention medical education only as a broad contextual element without in-depth analysis of AI implementation processes,or include medical education as a subject matter but do not specifically concentrate on it are excluded.

### Study selection and data abstraction

After removing the duplicate studies, two reviewers (Li and Yan) independently screened titles/abstracts and assessed full texts using predefined criteria. A pilot calibration (50 random articles, κ = 0.85) ensured initial consistency. All decisions were validated in duplicate, with disagreements resolved through team discussions and adjudication by a senior researcher (Lai), following PRISMA guidelines.

### Collating and summarizing data

Data collection and organization are achieved through a pre-designed table, including the first author, publication date, country, language, journal/periodical, title, document type, research topic, current ethical challenges of integration of AI and large language models in medical education, and suggestions to address these ethical challenges etc. Data collection is completed by two independent researchers, assisted by KIMI AI, who screen and cross-check the data. Disputed content was resolved through group discussion.

### Use of AI tools

(1) Names of AI tools used: We utilized Kimi AI to assist in various aspects of our research. (2) Description of how the tools were used: Kimi AI was employed to facilitate the initial screening of literature and extraction of key information, such as authors, publication dates, research topics, and main findings. It also assisted in generating preliminary outlines for sections of the manuscript and suggesting relevant research questions. (3) Evaluation of the validity of the tool’s outputs: To ensure the accuracy and reliability of the information generated by Kimi AI, we implemented a rigorous review process. Two independent researchers cross-checked the AI-generated content against the original sources. Any discrepancies or uncertainties were resolved through group discussions, ensuring that all data and information included in the study were thoroughly validated [17,18]. The complete record file of the use of Kimi AI can be found in S3 File.

### Critical appraisal within sources of evidence

The main objective of this review was to offer a comprehensive summary of the current literature and to integrate information for a broader understanding of the subject rather than performing an in-depth critical evaluation of individual studies. As such, we did not carry out a critical assessment of the evidence sources. The decision not to critically appraise the selected articles stemmed from it not being a goal of this review and would not provide any further significant insights [19].

## Results

### Study selection

**Fig 1:**
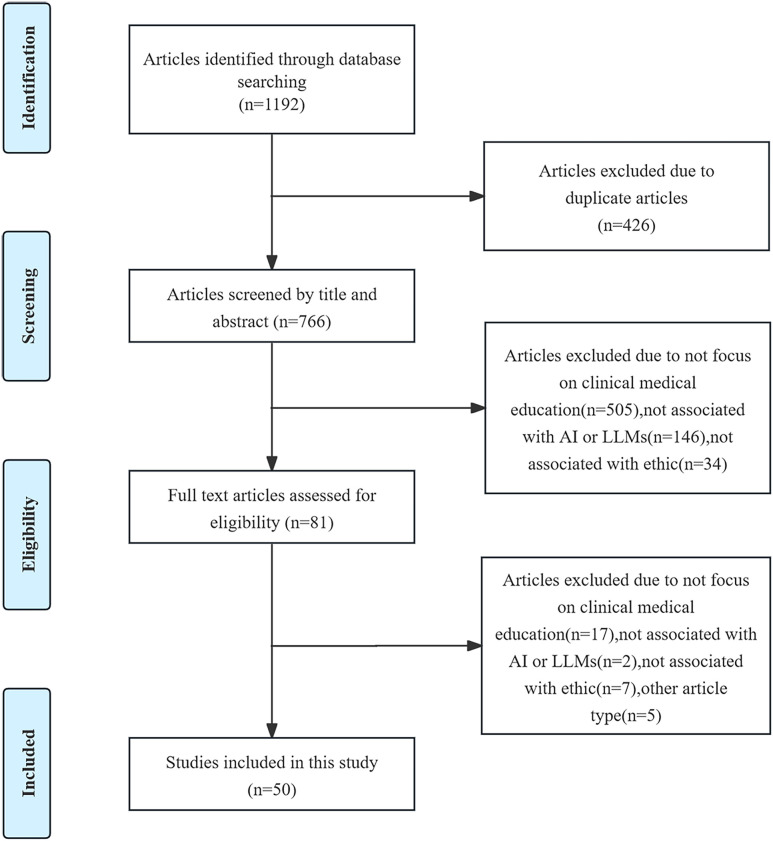
The PRISMA flow diagram for this study. PRISMA flow diagram summarizing the study’s article selection process. A total of 1192 articles were identified. Finally, 50 studies were included.

We identified 1192 relevant literature records from the database, excluded 426 duplicate records, and excluded 685 literature records by reading the titles and abstracts. Then, we excluded another 30 literature records by reading the full texts, and finally, 50 literature records were included in the study [6,13, 20–66]. The PRISMA flow diagram for this study is shown in Fig 1. All the data are presented in S1Table.

### Characteristics of the sources of evidence

**Fig 2:**
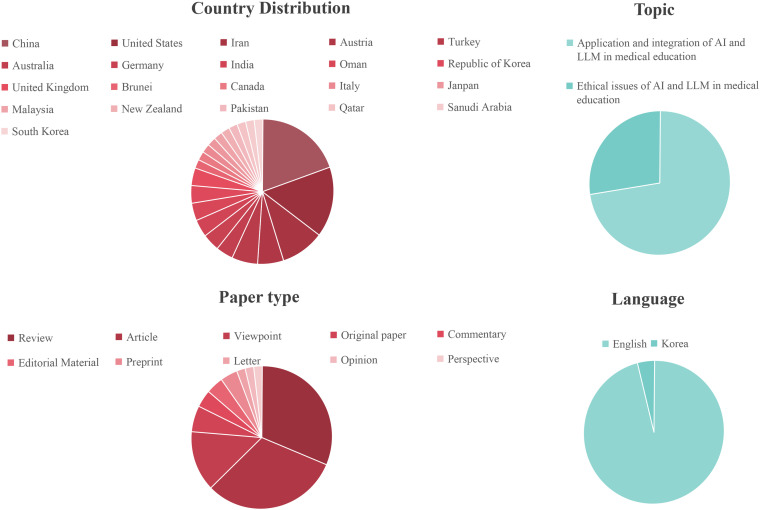
Distribution of included studies by country, language, type, and topic.

The summary of the included articles in our scoping review highlights the distribution and characteristics of the evidence base. Regarding geographical distribution, most papers were published in China and the United States, with 10 (20.00%) and 7 (14.00%) articles respectively. Other countries making notable contributions include Iran with 5 articles (10.00%), and Austria, Turkey, Australia, Germany, India, and Oman, each contributing 3 articles (6.00%). The distribution of publications across the years in our scoping review reveals a marked increase in scholarly output. There was a mere 1 publication (2.00%) in 2021, which increased to 4 publications (8.00%) in 2022, and significantly peaked at 24 publications (48.00%) in 2023, followed by 21 publications (42.00%) in 2024, indicating a substantial and recent focus on the integration of AI and LLM in medical education. A minority of the articles originated from a diverse range of other countries, each with a single publication, which is not detailed due to their low frequency. English was the predominant language of publication, with 48 articles (96.00%) written in English, followed by Korean with 2 articles (4.00%). This emphasizes the international nature of the discourse on AI and LLM in medical education. When examining the types of articles, a near-equal division was observed between reviews (15, 30.00%) and articles (16, 32.00%). Viewpoints accounted for 7 articles (14.00%), while original papers, commentaries, editorial materials, and preprints contributed a smaller proportion. The remaining categories, including letters, opinions, and perspectives, each accounted for a single article (1.96%). A total of 50 articles were considered, with a significant majority focusing on the application of AI and LLM in medical education, specifically highlighting the potential applications or benefits in 37 articles (74%) and the ethical issues in 13 articles (26.00%). This overview of the characteristics of the evidence sources provides a structured summary of the literature included in our scoping review, offering insights into the current scope and focus of research on AI and LLMs in medical education. The study characteristics are shown in Table 2 and Fig 2. S2 Table is the standardized study characteristics table.

**Table 2:** Distribution of included articles by country, year, language, type, and topic.

Summary of included articles	No, (% of 50)
***Country***	
China	10, (20.00%)
United States	7, (14.00%)
Iran	5, (10.00%)
Austria	3, (6.00%)
Turkey	3, (6.00%)
Australia	2, (4.00%)
Germany	2, (4.00%)
India	2, (4.00%)
Oman	2, (4.00%)
Republic of Korea	2, (4.00%)
United Kingdom	2, (4.00%)
Brunei	1, (2.00%)
Canada	1, (2.00%)
Italy	1, (2.00%)
Japan	1, (2.00%)
Malaysia	1, (2.00%)
New Zealand	1, (2.00%)
Pakistan	1, (2.00%)
Qatar	1, (2.00%)
Sanudi Arabia	1, (2.00%)
South Korea	1, (2.00%)
***Publication year***	
2021	1, (2.00%)
2022	4, (8.00%)
2023	24, (48.00%)
2024	21, (42.00%)
***Language***	
English	48, (96.00%)
Korea	2, (4.00%)
Paper type	
Review	16, (32.00%)
Article	15, (30.00%)
Viewpoint	7, (14.00%)
Original paper	3, (6.00%)
Commentary	2, (4.00%)
Editorial Material	2, (4.00%)
Preprint	2, (4.00%)
Letter	1, (2.00%)
Opinion	1, (2.00%)
Perspective	1, (2.00%)
***Topic***	
Application of AI and LLM in medical education	37, (74.00%)
Ethical issues of AI and LLM in medical education	13, (26.00%)

### Overview of AI and LLM applications in medical education

In a review of 50 selected articles, AI and LLMs’ most frequently discussed applications in medical education include their ability to provide personalized learning plans and feedback on student’s strengths and weaknesses, thereby enhancing learning efficiency and quality (84%). AI technology also plays a crucial role in simulating patient interactions and clinical scenarios, which supports the improvement of students’ diagnostic and interpersonal skills (75%). Furthermore, AI and LLMs foster interactive learning by providing dynamic, immersive educational experiences that replicate clinical environments (63%). Additionally, AI demonstrates significant value in facilitating simulated dialogues, intelligent tutoring, and automated Q&A functions, all of which aid in problem-based learning (PBL), team-based learning (TBL), case-based learning (CBL), and precision medical education. In research support, AI and LLMs have proven instrumental by automating literature reviews, offering research design recommendations, and conducting statistical analyses, thus contributing substantially to medical research [63–65].

### Ethical challenges and recommendations

**Fig 3:**
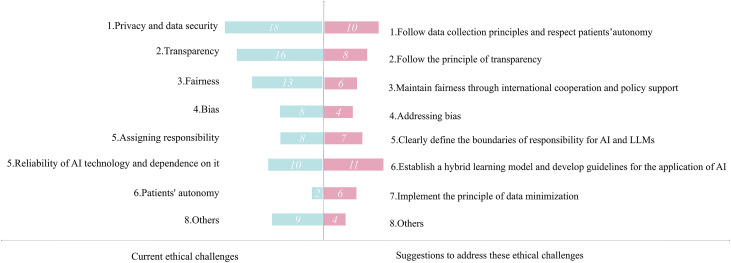
Summary of current ethical challenges and suggestions to address these challenges. The bar chart differentiates between two categories: current ethical challenges (represented by pink bars) and recommendations to address these challenges (represented by blue bars).The length of each bar corresponds to the number of studies discussing each item, with the exact count explicitly labeled within each bar for clarity. **Current ethical challenges.**

Our review of the same corpus of literature also revealed a complex web of ethical considerations arising from the integration of AI and LLMs in medical education, as summarized in Fig 3. The following sections provide a detailed qualitative analysis of each major ethical theme.

#### Privacy and data security.

Privacy and data security are the most prominent ethical challenges when AI and LLMs are applied in medical education. Given the extensive utilization of these technologies, safeguarding the sensitive health information of both students and patients is of paramount significance. Ensuring data confidentiality and preventing unauthorized access and misuse is a crucial concern [21,22,33,34,41,42,44,46,48,50,54]. The personalization of services and algorithm enhancements by AI and LLMs necessitate vast quantities of personal data, giving rise to issues related to privacy breaches and data security [59]. For instance, Rodriguez Peñaranda N’s study [67] emphasize that AI applications in surgical training, particularly for kidney cancer surgeries, entail extensive data collection, encompassing patient and procedural data. Additionally, the storage and transmission of patient examination data, such as ultrasound images, on AI platforms might expose vulnerabilities, as noted by Koçer Tulgar Y’s study [37]. These challenges are exacerbated by risks such as hacking, system breaches, or insufficient encryption measures, resulting in unauthorized access or data interception during transmission [60]. Data collection, frequently carried out without users’ awareness or explicit consent, presents specific risks when dealing with patient data or student information in educational settings [45,60]. The limited accessibility of regulated datasets and the absence of mechanisms to verify data authenticity further blur the distinctions between legitimate and unethical data usage. As pointed out by Gurnani B [32], AI may not fully comprehend the context of a problem and the subtle distinctions in ophthalmology cases, which could lead to privacy and data security issues.

#### Transparency.

The lack of transparency in AI decision-making processes, commonly referred to as “black box algorithms,” poses a significant challenge to the application of artificial intelligence and large language models in medical education [22,28]. This issue has been extensively discussed in the included literature, with various studies highlighting the problem of opacity in AI systems from multiple dimensions [22,28,35,38–40,42,45,48,55,57–59,62,66,68]. The core problem lies in the inherent inability of users to comprehend or observe the internal decision-making logic of these systems, which not only undermines trust but also raises concerns regarding the accuracy and reliability of their outputs [35,40,45,59]. Ethical concerns extend beyond technical opacity to include lack of accountability [39,58], ambiguities in the copyright of AI-generated content [48], and algorithmic biases stemming from non-transparent training data [42,57]. Particularly in the field of medical education, the lack of explainability in AI decision-making processes raises critical questions about how these systems reach conclusions that may impact student assessment, curriculum design, and ultimately patient care [35,45,57,59].The “black box” nature of these algorithms not only exacerbates distrust in AI systems [20,40,44,59] but also creates significant challenges for validating educational outcomes and ensuring ethical implementation across diverse learning environments [44,47,52,59,62,68]. This comprehensive lack of transparency necessitates the development of more explainable AI approaches and underscores the urgent need for ethical guidelines specifically addressing algorithmic transparency in medical education contexts [27,41,44,45].

#### Fairness.

The application of AI and LLM in medical education has drawn extensive attention to issues concerning fairness, as there are concerns that AI technology might intensify unequal resource allocation [59], particularly between high-income countries (HICs) and low-income and middle-income countries (LMICs) [26]. In the realm of medical education, the uneven distribution of medical resources, economic imbalances, technological divides, and variations in teaching standards could aggravate educational inequity [27,32,43,44,46,51,52,54,59,64,46,69]. Furthermore, the inherent bias in AI and LLM may also exacerbate unfairness towards specific groups and impact the quality of medical education [28]. For instance, due to its incapacity to consider cultural diversity in its algorithms, students from diverse cultural backgrounds may undergo unfair education [30,50]. One study has also discovered that the application of AI in ophthalmology education might be influenced by data bias, resulting in unfair services for certain groups of people [32].

#### Bias.

The reliance upon non-representative training data and inherent biases in data collection can give rise to discriminatory decisions against specific patient groups [24,39,48,59,61]. For example, [67] disclose that biases in surgical training data might lead to imprecise skill evaluations, while Vahedifard [70] emphasizes disparities in mental health services attributed to biased AI algorithms [44]. These issues highlight the risks of unequal treatment and diagnosis that originate from biased datasets and opaque systems. Additionally, Walsh, Stogiannos [57] have indicated that the deficiency of transparency and bias in AI within medical imaging can impact the accuracy and fairness of diagnoses.

#### Responsibility and accountability.

Assigning responsibility for errors in AI-driven medical decisions is a major ethical concern. The legal and moral responsibilities of virtual agents like ChatGPT in healthcare remain ambiguous [20,26,46,47,52,58,64].In the context of medical education, presents challenges across different dimensions: on one hand responsibility allocation might encompass issues like plagiarism and academic misconduct, on the other hand stemming from emerging uncertainties in technical guidance and application [27]. The research conducted by [67] suggests that the concept of responsibility may become blurred when AI technology is utilized for assessing surgical skills. Specifically, where AI systems provide erroneous assessments or misleading guidance, this tends to blur the demarcation among liable parties.

#### Reliability and dependence.

The questionable reliability of AI itself and excessive reliance on AI tools may weaken students’ critical thinking and problem-solving skill [39,51,55]. Dependence on AI-generated answers might impede students’ ability to independently handle complex medical issues [63]. Relying on AI tools also increases the risks of academic misconduct, such as plagiarism [53,64,68]. Research has indicated that the “hallucinations” of AI and LLM, in which the system generates or provides false or inaccurate information or data in the absence of appropriate information and may present fabricated references due to the inability to provide specific sources, have the potential to mislead medical professionals and medical students in medical education and clinical decision-making [40]. The constraints of current AI models and the quality of data also prompt people to query the reliability of the decisions they generate.For example, AI models trained on biased or outdated data may propagate distorted or erroneous cultural perspectives and clinical guidelines, particularly when applied to medical contexts beyond their original training scope. Furthermore, in complex clinical cases, analyses and diagnoses generated by AI and LLMs may lack comprehensiveness, potentially overlooking critical nuances [58,67].

#### Patient’s autonomy and consent.

With the incorporation of AI in medical education and healthcare, patients’ autonomy is confronted with predicaments. Regarding the collection of a large amount of patients’ data, the collection and management of such data might encroach upon patients’ autonomy [45,57]. The use of AI technology in medical education and healthcare may lead medical students to face a dilemma between relying solely on AI diagnostic recommendations and considering the patient’s symptoms and specific circumstances. Whether the patient has an exhaustive understanding of AI and whether they are informed and consent to the decision directly impacts the patient’s right to make an informed decision regarding their medical needs [45,57].

#### Other ethical challenges.

In addition, academic integrity and plagiarism issues, technology misuse and manipulation issues, cultural sensitivity and diversity issues, potential for malicious use, and technology-induced employment issues are also ethical challenges that need to be considered [13,20,27,31,52,53,60–62].

### Risk mitigation strategies for ethical challenges

#### Implementing privacy protection measures.

Concerning privacy protection and data security, it is crucial to consider the issues of data privacy and security during the design and implementation of AI and LLM systems employed in medical education and embrace corresponding protective measures [20,33,35,42,46,67]. It is essential to establish agreements with relevant institutions to clarify, justify, implement, and oversee the necessity of both proactive and passive data collection, and to enhance the ethical review requirements of institutional review boards (IRBs) and national institutions to ensure their accommodation to the advancement of the AI data collection process [45]. Regulatory measures and statutes should be formulated and executed to supervise the authenticity of data and data abuse conducts, such as the EU’s GDPR prohibiting automated decision-making and data processing [60]. It is crucial to safeguard the privacy of patients and students during data processing through data encryption, data anonymization, the implementation of secure storage protocols, and the enforcement of vigorous access control [50]. For instance, in medical education settings, educators should aim to minimize the use of actual patient data in AI and LLMs and prefer to use synthetic or hypothetical scenarios to avert any potential privacy breaches. In cases in which it is necessary to input patient-specific information into AI and LLMs, it is imperative to obtain informed consent from the patient, obtain the necessary approval from an ethics committee, and implement appropriate data anonymization measures. Additionally, it is vital to provide medical students with comprehensive education and training regarding patient privacy protection, including lawful and ethical procedures for the collection, use, and storage of patient information. This foundation is crucial for fostering robust privacy awareness as AI and LLMs become increasingly pervasive in medical education. [20,33,35,42,46,67]. In the long term, AI ethics education can be incorporated in medical education to ensure that future healthcare professionals not only apprehend the ethical dimensions of AI in medicine but also are competent to make ethical decisions in practice [59].

#### Promoting transparency.

When developing and deploying AI models, strict adherence to the principle of transparency is indispensable, facilitating the comprehensibility and verifiability of the decision-making process and outcomes for users [22,46]. One study in2024 presented a detailed AITEL report checklist, stipulating detailed information concerning algorithm design, training data, testing/validation, etc. when reporting on AI systems to enhance transparency. Collaboration among computer scientists, medical practitioners, and ethicists should be encouraged to address the issue of algorithm opacity through multi-disciplinary cooperation. The incorporation of AI ethics education in medical education is requisite to ensure that future healthcare professionals can comprehend and handle such ethical matters [25,39,61,62].

#### Ensuring fairness through policy and cooperation.

To safeguard the equity of medical education in various regions and cultural milieus, international cooperation and policy support can be harnessed to aid regions endowed with limited resources in acquiring and applying AI technology. Policymakers should prioritize resource allocation and control data-sharing protocols to ensure equitable access to AI technologies [22,27,56,57]. A study suggested that through the provision of fundamental technologies and the reinforcement of AI education, it could be guaranteed that the utilization of AI technology in LMICs could satisfy local requirements [26]. When developing and utilizing AI applications, it is essential to create algorithms to identify and eliminate potential unfair factors, and adapt to different cultural backgrounds to ensure the targeted and sensitive use of AI tools [20].

#### Addressing bias.

Diverse datasets and algorithms should be employed in the training of AI algorithms, guaranteeing the representativeness and comprehensiveness of the data to uphold transparency for diverse patient groups and alleviate bias [28,59,67]. Regular bias audits should be carried out to identify and rectify biases present in AI systems [28].

#### Defining responsibility and accountability in AI and LLMs.

Specify the delimitation of responsibility for AI and LLM systems, especially in predictive and decision-making functions [45,54]. Enact laws and regulations to clarify liability for AI-related errors [41]. Instructing medical students on responsibility and accountability, while implementing rigorous evaluation and oversight mechanisms to oversee the use of AI, especially in cases where incorrect predictions lead to patient harm [22,28,32]. Highlight the role of instructors in guiding students on the proper and ethical application of AI tools.

#### Developing a hybrid learning model and AI application guidelines.

Adopt Embrace hybrid educational paradigms integrating AI tools with traditional teaching methodologies, stimulating students’ independent thinking and problem-solving capabilities [27,28,60]. Formulate guidelines for applying AI and LLMs in medical education and educate students about the constraints of AI to alleviate excessive reliance [32,33,56–58,62,66]. Elevate data quality, diversify data collections, and augment model transparency and interpretability to tackle AI “hallucinations” and unreliable outputs [63]. Stress human supervision and feedback mechanisms in the designs of AI systems to guarantee accuracy and reliability.

#### Respecting patient autonomy and data collection principles.

To implement the principle of data minimization and enhance patient autonomy in medical decision-making, we must collect only the essential minimum data required to achieve medical objectives, thereby reinforcing data security throughout the collection process [22,23,37,49]. Before data collection, it is crucial to elucidate the scope and purpose of data gathering to patients, ensuring their informed consent and understanding [22]. Additionally, enhancing the transparency and interpretability of AI algorithms will facilitate patients’ comprehension of the AI system’s decision-making process, which is vital for maintaining trust and autonomy [13]. Concurrently, intensifying education on respecting patient autonomy within medical education will equip future healthcare professionals with the ethical considerations necessary to balance technological advancements with patient rights [13,36].

#### Other risk mitigation strategies.

Besides the six suggestions stated above, structured training of AI in medical education, critical use of AI technologies, comprehensive reporting of AI system datasets, and explicit policies on academic misconduct can potentially mitigate or address the ethical challenges encountered when integrating AI and LLM in medical education [34,36,42,46].

## Discussion

### Summary of evidence

Our analysis of 50 articles reveals that the ethical landscape of AI/LLM integration in medical education is not merely a list of discrete issues but a synergistic system of tensions and trade-offs, encompassing key challenges such as privacy and data security, transparency, fairness, bias, responsibility and accountability, reliability and dependence, and patient’s autonomy and consent.

The most quantified challenge, data privacy and security (Fig 3), transcends its frequency to act as a core foundational barrier [71]. The primary ethical conflict here is not just about preventing breaches, but the philosophical dilemma of submitting sensitive, identifiable patient data into proprietary systems whose data usage protocols remain opaque [72,73]. This creates a fundamental value conflict with algorithmic transparency, as stringent privacy protections inevitably hinder external auditing for model bias and fairness [74], thereby challenging clinical acceptance and ethical deployment. For example, Rajkomar et al. argue that without access to training data and model weights, it is nearly impossible to fully diagnose and mitigate discriminatory outputs [75]. The ethical tensions between privacy and transparency directly intensify the third core challenge: fairness in global medical education. The opacity of AI systems impedes the detection of data and algorithmic biases, which can exacerbate existing global disparities and marginalize underrepresented student populations [76,77]. Beyond these core tensions, our review also identified additional intertwined challenges, including questions of responsibility and accountability, the reliability of AI outputs and the risk of over-dependence, and threats to patient autonomy and consent in data collection practices. These issues do not exist in isolation but interact with and compound the primary challenges of privacy, transparency, and fairness, reinforcing the notion of a complex ethical ecosystem.

### Preliminary ethical comparison between AI and LLMs in medical education

In this study, we conducted a preliminary comparative analysis of the ethical challenges associated with the use of artificial intelligence (AI) and large language models (LLMs) in medical education. Our findings indicate that while both technologies face overarching ethical concerns—such as privacy and data security, transparency, bias, accountability, fairness, reliability, dependence, and patient autonomy—their manifestations and degrees of emphasis differ.

Notably, traditional AI applications frequently handle sensitive and identifiable patient data, which leads to more pronounced challenges pertaining to privacy and data security [78]. Furthermore, such applications often raise greater concerns regarding fairness, as they are commonly associated with biased algorithmic outcomes in assessment and resource allocation [79]. In contrast, LLMs, due to their generative nature and potential for “hallucinations”, attract more attention concerning reliability and over-dependence [68]. Additionally, owing to the opacity inherent in their automated content generation processes, LLMs also present significant challenges in transparency and accountability [80].

It should be noted that these distinctions are preliminary and based on emerging trends within the included literature. Further empirical and qualitative studies are needed to validate and refine these observations. Nonetheless, this comparative perspective offers an initial framework for understanding and addressing technology-specific ethical dilemmas in medical education, thereby aiding educators, developers, and policymakers in adopting more nuanced and effective governance strategies.

### Strengths and limitations

The strengths of this scoping review are diverse and highlight its essential contribution to the field. First, a systematic search strategy was utilized across PubMed, Embase, and Web of Science, ensuring a comprehensive literature capture and enhancing the review’s reliability. Second, including perspectives from various countries reflects a global dialogue on the ethical challenges posed by AI and large language models (LLMs) in medical education. Third, the review’s timely focus on the increase in scholarly outputs from 2021 to 2024 emphasizes the relevance of these issues in the continuously evolving landscape of medical education. Fourth, many articles’ structured analysis of ethical challenges provides a clear overview of the considerations involved in integrating AI and LLMs. Lastly, the review presents concrete solutions and recommendations, offering actionable guidance for future research and policy formulation regarding the ethical application of AI in medical education. These strengths establish the review as a solid foundation for advancing the ethical discourse and practical application of AI in this field.

After reviewing the existing literature, we identified several potential limitations that may affect our understanding of AI and LLMs’ application and ethical issues in medical education. Firstly, 96.08% of the articles were in English, which could introduce bias in our perspectives, particularly regarding insights from non-English-speaking regions. Secondly, our inclusion criteria may have unintentionally excluded relevant resources, as they required articles to explicitly discuss the application of AI and LLMs in medical education or related ethical issues. Additionally, including various studies (e.g., reviews, articles, perspectives) could lead to inconsistencies in evidence quality and rigor, impacting the integration and interpretation of research findings. Lastly, while we aim for methodological rigor, selecting articles based solely on their “ethical challenges” discussion may still result in selection bias. Therefore, we must interpret these findings cautiously, recognizing the potential impact of these limitations on our results.

### Implications

The findings of this review hold significant implications for future research and policy development concerning the responsible utilization of AI and LLMs in medical education. This study’s principal importance underscores the necessity of establishing ethical frameworks and guidelines for the application of AI within this domain. Such measures are essential to ensure that the integration of these technologies is both practical and aligned with ethical standards.

To address the complex ethical landscape identified in the current study, several actions are recommended:

(1) Developing differentiated ethical guidelines: Policymakers and institutions should prioritize the creation of context-specific guidelines that differentiate between the ethical risks of traditional AI tools and those inherent to generative LLMs [81]. For instance, guidelines for LLMs must specifically address accountability for “hallucinations” and require transparency in training data sources, whereas policies for adaptive learning platforms might focus more on data sovereignty and algorithmic fairness in assessment [82]. Furthermore, there is an urgent need for clarifying legal liability [20] through legislative action, as the current ambiguity stifles innovation and adoption [83].
(2) Integrating ethics into curriculum and faculty development: For medical educators and institutions, our synthesis implies that ethical competency must become a core component of digital health literacy. Curricula should be updated to include critical appraisal of AI-generated content, understanding of data privacy principles, and awareness of algorithmic bias [20]. The proposed “hybrid learning model” should be operationalized to position AI as a critical tool for augmentation rather than replacement, ensuring that human oversight and professional judgment remain central to clinical reasoning [84]. Institutions investing in AI tools must couple this investment with comprehensive faculty development programs focused on the ethical pedagogical use of these technologies.
(3) Focusing future research on critical gaps: This review highlights critical gaps that demand targeted future inquiry. Future research should employ qualitative methods (e.g., deliberative workshops) to explore the ethical trade-offs stakeholders are willing to make (e.g., trading some privacy for greater educational efficacy) and to validate the preliminary differentiation between AI and LLM ethics proposed here [85]. Furthermore, rather than merely describing the problem, interventional studies are needed to develop and test models for equitable AI access and implementation in low-resource settings, moving from theory to action. The complex interplay of challenges requires deep interdisciplinary collaboration; future work should co-create solutions with teams comprising ethicists, clinicians, data scientists, lawyers, and patients to ensure they are robust, practical, and ethically sound. Lastly, research must move beyond conceptual analysis to longitudinal studies that track the real-world ethical outcomes of AI integration in medical education over time.

This review provides a foundational framework for future research and policy-making, fostering advancements in medical education practices, promoting global health equity, considering long-term ethical considerations, and enhancing interdisciplinary collaboration. We can strive toward a more moral and practical integration of AI and LLMs in medical education by addressing the identified challenges and adhering to the proposed measures and research directions.

## Conclusion

Medical ethics principles are fundamentally constituted by four core tenets: respect for autonomy, beneficence, non-maleficence, and justiceThe integration of AI and LLMs into medical education presents a dualistic landscape of immense potential and significant ethical complexity. Our scoping review identifies a core tension: these technologies risk undermining the very ethical principles they aim to teach, particularly respect for autonomy (through privacy violations and opaque decision-making) and justice (through perpetuated algorithmic biases). The defining mission of medical education(to cultivate knowledgeable, skilled, and ethically-grounded practitioners) demands that this integration be guided not solely by technological capability but by a steadfast commitment to medical ethics. Therefore, AI and LLMs should not be viewed merely as tools for efficiency, but as catalysts for developing deeper ethical reasoning and digital literacy among future healthcare professionals. Moving forward, the field must transition from conceptual mapping to actionable solutions. This necessitates robust empirical research to evaluate the real-world impact of AI on learning and ethical development, a dedicated focus on ensuring these technologies promote rather than hinder global health equity, and a critical examination of their long-term implications for healthcare. Ultimately, the goal is a future where AI integration strengthens, rather than compromises, the foundation of ethical medical practice.

## Supporting information

S1 File The PRISMA-ScR checklist. (DOCX)

S2 File The specific search terms for each database. (DOCX)

S3 File Detailed Records of AI Usage. (DOCX)

S1 Table Summary of Studies Identified and Screened in the Literature Search. (XLSX)

S2 Table The standard characteristics of study table. (XLSX)
