# Supplementary material for: The ethical challenges in the integration of artificial intelligence and large language models in medical education: A scoping review
Source: PLoS One. 2025 Oct 22;20(10):e0333411. doi: 10.1371/journal.pone.0333411 (PMC12543126; doi:10.1371/journal.pone.0333411)
Supplement: S2 File — (DOCX) [file pone.0333411.s002.docx]

**S2 file. The specific search terms for each database.**

**1. PubMed:**

(((Medical[Title/Abstract] OR Medicine[Title/Abstract]) AND (Education[Title/Abstract] OR Educate[Title/Abstract])) AND (Artificial Intelligence[Title/Abstract] OR AI[Title/Abstract] OR Machine Learning[Title/Abstract] OR ML[Title/Abstract] OR Deep Learning[Title/Abstract] OR Data Analytics[Title/Abstract] OR Natural Language Processing[Title/Abstract] OR Neural Networks[Title/Abstract] OR Pattern Recognition[Title/Abstract] OR Data Mining[Title/Abstract] OR Computer Vision[Title/Abstract] OR Reinforcement Learning[Title/Abstract] OR Automated Reasoning[Title/Abstract] OR Cognitive Computing[Title/Abstract] OR Machine Intelligence[Title/Abstract] OR Intelligent Systems[Title/Abstract] OR Intelligent Control[Title/Abstract] OR Large language Model[Title/Abstract] OR LLM[Title/Abstract] OR Natural Language model[Title/Abstract] OR NLM[Title/Abstract] OR Chatgpt[Title/Abstract] OR multimodal[Title/Abstract] OR multimodality[Title/Abstract])) AND (Ethical[Text Word] OR Ethic[Text Word]) Filters: from 2011 - 2024 Sort by: Most Recent

**2. Embase**

(medical:ti,ab,kw OR medicine:ti,ab,kw) AND (education:ti,ab,kw OR educate:ti,ab,kw) AND ('artificial intelligence':ti,ab,kw OR ai:ti,ab,kw OR 'machine learning':ti,ab,kw OR ml:ti,ab,kw OR 'deep learning':ti,ab,kw OR 'data analytics':ti,ab,kw OR 'natural language processing':ti,ab,kw OR 'neural networks':ti,ab,kw OR 'pattern recognition':ti,ab,kw OR 'data mining':ti,ab,kw OR 'computer vision':ti,ab,kw OR 'reinforcement learning':ti,ab,kw OR 'automated reasoning':ti,ab,kw OR 'cognitive computing':ti,ab,kw OR 'machine intelligence':ti,ab,kw OR 'intelligent systems':ti,ab,kw OR 'intelligent control':ti,ab,kw OR 'large language model':ti,ab,kw OR llm:ti,ab,kw OR 'natural language model':ti,ab,kw OR nlm:ti,ab,kw OR chatgpt:ti,ab,kw OR multimodal:ti,ab,kw OR multimodality:ti,ab,kw) AND (ethical OR ethic) AND [01-01-2011]/sd AND [01-01-2001]/sd NOT [01-09-2024]/sd

**3. Web of Science**

Medical OR Medicine (Topic) and Artificial Intelligence OR AI OR Machine Learning OR ML OR Deep Learning OR Data Analytics OR Natural Language Processing OR Neural Networks OR Pattern Recognition OR Data Mining OR Computer Vision OR Reinforcement Learning OR Automated Reasoning OR Cognitive Computing OR Machine Intelligence OR Intelligent Systems OR Intelligent Control OR Large language Model OR LLM OR Natural Language model OR NLM OR Chatgpt OR multimodal OR multimodality (Topic) and Ethical OR Ethic (Abstract) and Education OR Educate (Topic) Timespan: 2010-01-01 to 2024-08-30 (Publication Date)
