# Supplementary material for: The ethical challenges in the integration of artificial intelligence and large language models in medical education: A scoping review
Source: PLoS One. 2025 Oct 22;20(10):e0333411. doi: 10.1371/journal.pone.0333411 (PMC12543126; doi:10.1371/journal.pone.0333411)
Supplement: S3 File — (DOCX) [file pone.0333411.s003.docx]

**S3 file. Detailed Records of AI Usage.**

In the process of extracting information from the literature, we utilized KIMI AI to streamline the procedure. Specifically, we uploaded the PDF versions of the 50 eligible articles that met our inclusion criteria into the KIMI AI platform. The tool was then employed to extract the required information, including the country of the first author, the language of the article, the publication date, and the type of the article. Additionally, KIMI AI was used to summarize the applications of artificial intelligence and large language models in medical education and training, as well as to identify any ethical challenges or issues mentioned in the articles. To ensure the comprehensiveness and accuracy of the extracted information, we conducted a thorough manual review of the full texts of the articles. This step allowed us to verify the information extracted by KIMI AI and to cross-check for any additional details that may have been overlooked, particularly those related to ethics that might be present in figures and tables.

All articles were analyzed using the following prompt: “Please extract the country of the first author, the language of the article, the publication date, and the type of the article (whether it is an Article, Review, Preprint, or other type). Summarize the applications of artificial intelligence/large language models in medical education/training mentioned in the article. Identify any ethical challenges, difficulties, or issues related to ethics mentioned in the article. Also, provide any suggestions or solutions proposed to address these ethical challenges. Please ensure that any information related to ethics in the figures and tables is not overlooked and is extracted accordingly.”

User:

•1.Integrating AI in medical education: embracing ethical usage and critical understanding.pdf

Kimi:

First Author’s Country: Brunei

Article Language: English

Article Publication Date: 13 October 2023

Article Type: Opinion

Applications of AI/Large Language Models in Medical Education/Training:

•Personalized recommendations

•Improvement in medical diagnostics, especially in medical imaging

•AI chatbots for education, such as Khanmingo, to supplement and personalize student education

•AI virtual environments, AI-based assessments, and adaptive e-learning systems

•Learner-oriented AI tools for students to receive and understand new information

•Instructor-oriented AI to help teachers reduce workload and gain insights about students

•Institution-oriented AI for decision-making in managing and administrating schools

Ethical Challenges/Difficulties/Issues:

•Legal liability and accountability in clinical decisions made with AI support

•Concerns about who is responsible in the event of AI errors in clinical decision support systems

•The potential for AI to compromise critical thinking skills and scholarly integrity

•The phenomenon of "hallucination," where artificial intelligence generates non-existent references.

•Superficial engagement with knowledge without critical understanding

Suggestions/Solutions for Ethical Challenges:

•Develop interpretable generative AI algorithms that explain the AI decision-making process

•Use encryption technology, access control mechanisms, and data anonymization, and de-identification to address data privacy and security

•Develop algorithms that can identify potentially unfair factors and eliminate them while still meeting the needs of the students to address personalization and fairness

•Improve the quality and quantity of data by including diverse data while ensuring quality and consistency of labeled data to enhance effectiveness and reliability

User:

•2.Artificial intelligence takes center stage: exploring the capabilities and implications of ChatGPT and other AI-assisted technologies in scientific research and education.pdf

Kimi:

First Author’s Country: Australia

Article Language: English

Article Publication Date: 2023

Article Type: Review

Applications of AI/Large Language Models in Medical Education/Training:

•AI-assisted technologies to bridge language barriers in scientific research, improving accessibility and facilitating collaboration.

•Use of ChatGPT as a writing assistant to clarify convoluted text, simplify jargon, and improve the readability of scientific literature.

•Assistance for non-native English speakers in science to improve their communication skills and pronunciation.

•Support for thesis writing for students and supervisors, potentially reducing the editing load.

•Aid in organizing conference programs, although with limitations in generating specific and accurate content.

•Assistance with data analysis, coding, and interpretation in bioinformatics, empowering bench scientists to analyze their own datasets.

•Assistance with complex image analysis, potentially accelerating the generation of bespoke coding packages.

Ethical Challenges/Difficulties/Issues:

•Concerns about bias and accuracy in text generation, particularly in scientific research where accuracy and objectivity are paramount.

•Risks of AI-generated misinformation and the reliance on generative AI potentially undermining critical skills.

•Legal implications, including the use of content shared in queries or prompts for training generative models without restrictions on future use.

•Privacy concerns and potential data breaches, including the posting of personal data onto the platform.

•Copyright ownership and moral rights’ infringement, with questions about the use of human-generated works to train or inform AI tools.

•Safety and security concerns, including the potential misuse of generative AI for creating deepfakes and the spread of misinformation.

•The definition and measurement of intelligence in the context of AI and the implications of AI’s rapid adoption and capabilities.

Suggestions/Solutions for Ethical Challenges:

•Ensure AI is aligned with goals that promote human flourishing through alignment research and the application of behavioral ethics.

•Be cautious and vigilant in what is posted on AI platforms, scrutinizing outputs for accuracy and legality.

•Address legal concerns by understanding the implications of using AI tools for law-related services and the unauthorized practice of law.

•Stay updated on AI-based cyber threats and prioritize data security and privacy.

•Reflect on the meaning of intelligence in the context of AI and prepare for potential future scenarios, including the possibility of AI sentience.

User:

3.Biomedical Ethical Aspects Towards the Implementation of Artificial Intelligence in Medical Education.pdf

Kimi:

First Author’s Country: Germany

Article Language: English

Article Publication Date: 7 June 2023

Article Type: Commentary

Applications of AI/Large Language Models in Medical Education/Training:

•Virtual patient simulations or case studies for practicing diagnoses and treatments.

•Machine learning for image analysis to interpret medical images and detect anomalies.

•Natural language processing models for transcribing medical records or content.

•Tailored or intelligent learning plans and feedback based on personal strengths and weaknesses.

•Assistance in understanding and using AI concepts and their potential applications in clinical work.

Ethical Challenges/Difficulties/Issues:

•Lack of transparency and insufficient knowledge about AI applications.

•False/misleading results due to AI algorithms trained on biased data.

•Potential for perpetuating existing inequities in the medical field.

•Insufficient protection of data privacy and confidentiality.

•Risk of compromising patient autonomy and dignity.

•Challenges to user autonomy, including the generation of erroneous information by AI.

•Dependence on AI technology potentially hindering the development of essential decision-making skills.

Suggestions/Solutions for Ethical Challenges:

•Define learning purposes and objectives when using educational AI.

•Involve all relevant stakeholders in the decision-making process.

•Ensure transparency and comprehensibility in the use of AI in the medical curriculum.

•Obtain informed consent from users to ensure they understand and agree to use AI applications.

•Promote professional responsibility and accountability.

•Offer AI models as an add-on to the medical curriculum, not a replacement for traditional teaching.

•Control data-sharing agreements and establish laws/regulations for fair data use in AI development.

•Establish an equity and social justice framework before integrating AI into medical education.

•Regularly audit AI tools to detect and correct potential biases and inequities.

•Educate all users about AI functionalities, known biases, and potential risks.

•Provide AI-independent information to enable informed decisions without reliance on AI.

User:

4.Artificial intelligence in medical education curriculum An e-Delphi study for competencies.pdf

Kimi:

First Author’s Country: Turkey

Article Language: English

Article Publication Date: July 21, 2022

Article Type: Research Article

Applications of AI/Large Language Models in Medical Education/Training:

•The study aims to identify the competencies required by medical graduates to be prepared for AI technologies and their applications in medicine. It does not specifically mention the use of AI or large language models in medical education but focuses on the competencies that would enable medical graduates to work effectively with AI in a medical context.

Ethical Challenges/Difficulties/Issues:

•The study discusses the importance of using health data in accordance with legal and ethical norms.

•It emphasizes acting in accordance with ethical principles while using AI technologies.

•The study also highlights the importance of keeping healthcare records in a format that AI can process, which raises issues of data privacy and security.

•There is a mention of the importance of data collection, analysis, evaluation, and safety in the development of AI applications in healthcare, which implies concerns about data ethics and the responsible use of AI.

Suggestions/Solutions for Ethical Challenges:

•The study provides a consensus list of competencies that include understanding and applying legal and ethical norms in the use of health data.

•It suggests that medical graduates should be trained to use AI technologies in a manner that adheres to ethical principles.

•The study implies that medical education should include training on data privacy and security to ensure that healthcare records are managed responsibly.

•It recommends that medical graduates should be aware of the importance of data safety and should be trained to contribute to the development of AI applications in healthcare in an ethically responsible way.

User:

•5.Artificial intelligence in medical education: a cross-sectional needs assessment.pdf

Kimi:

First Author’s Country: Turkey

Article Language: English

Article Publication Date: 2022

Article Type: Research Article

Applications of AI/Large Language Models in Medical Education/Training:

•The study examines the perceptions of future physicians on the possible influences of artificial intelligence on medicine.

•AI is perceived as an assistive technology that could facilitate physicians’ access to information and patients’ access to healthcare, and reduce errors.

•AI applications are also considered in terms of their potential to devalue the medical profession, damage trust, and negatively affect patient-physician relationships.

Ethical Challenges/Difficulties/Issues:

•Concerns about AI leading to unemployment and reducing the need for physicians.

•Potential for AI to devalue the medical profession and erode trust in patient-physician relationships.

•Risks of violations of professional confidentiality due to AI applications in medicine.

•Lack of competence among students to inform patients about the features and risks of AI.

Suggestions/Solutions for Ethical Challenges:

•There is a need for structured training on AI applications during medical education.

•Topics related to AI in medicine should be integrated into the medical curriculum, including knowledge and skills related to AI applications, applications for reducing medical errors, and training to prevent and solve ethical problems that may arise from using AI applications.

•The study suggests that medical students should be educated on the ethical dimensions of AI to ensure professional values and rights are protected.

User:

•6.Ethical concerns surrounding artificial intelligence in anatomy education Should AI human body simulations replace donors in the dissection room?.pdf

Kimi:

First Author’s Country: New Zealand

Article Language: English

Article Publication Date: 2023

Article Type: Article

Applications of AI/Large Language Models in Medical Education/Training:

•The article discusses the hypothetical replacement of human body donors with AI human body simulations in anatomy education.

•It considers the potential of AI to offer adaptive ability, real-time feedback, “smart” analytics, continuous improvement abilities, instant simulations, and potential for “intelligent” tutoring in the dissection room.

Ethical Challenges/Difficulties/Issues:

1.The uncertainty of the impact of replacing donors with AI simulations.

2.The risk of eroding the humanistic aspects of healthcare education and professional identity formation.

3.The potential loss of engagement with bioethical principles that are intertwined with the dissection room experience.

4.Concerns about the authenticity and human connection in education, which AI simulations may not be able to replicate.

Suggestions/Solutions for Ethical Challenges:

•Protect and maintain exposure to human body donors and dissection room time, rather than replacing them with AI simulations.

•Any move towards AI in anatomy education should be closely scrutinized, monitored, and transparently assessed to identify and mitigate adverse outcomes.

•Reflect on the dictum that healthcare and anatomy are uniquely human endeavors, and consider the implications of AI on these humanistic experiences.

User:

•7.Artificial intelligence and medical education A global mixed-methods study of medical students’ perspectives.pdf

Kimi:

First Author’s Country: United Kingdom Article Language: English Article Publication Date: Accepted date: 6 March 2022 Article Type: Original Research

Applications of Artificial Intelligence/Large Language Models in Medical Education/Training mentioned in the article: 1. Clinical applications of AI in medicine, including radiology, pathology, and oncology. 2. Algorithm development and coding. 3. AI ethics education. 4. Integration of AI in clinical practice. 5. Use of AI to improve access to care in underserved communities, particularly in low- and middle-income countries (LMICs).

Ethical Challenges or Difficulties or Issues mentioned in the article: 1. Interpretability of AI results and incorporation into clinical culture. 2. Trust in healthcare AI reduced by reports of systemic racial biases in algorithms. 3. Potential exacerbation of health inequalities due to the concentration and development of AI in high-income countries (HICs). 4. Concerns about AI replacing physicians and causing unintentional harm or discrimination due to data bias.

Suggestions or Solutions for these Ethical Challenges: 1. Education on AI among clinicians to ensure a patient-centered and equitable rollout of AI. 2. Teaching AI to those embedded within the systems that will use it, ensuring local applicability of AI tools. 3. Providing foundational technology and robust AI education in LMICs to drive innovation in healthcare settings. 4. Multidisciplinary education involving computer science students to address the ethical and technical aspects of AI in medicine.

English Text: First Author’s Country: United Kingdom Article Language: English Article Publication Date: Accepted date: 6 March 2022 Article Type: Original Research

Applications of AI in medical education as mentioned in the article include clinical applications in radiology, pathology, and oncology; algorithm development and coding; AI ethics education; integration of AI in clinical practice; and the use of AI to improve access to care in underserved communities, especially in LMICs.

Ethical challenges mentioned in the article include the interpretability of AI results, trust issues due to racial biases in algorithms, exacerbation of health inequalities, and concerns about AI replacing physicians or causing harm due to data bias.

Suggestions to address these ethical challenges include educating clinicians on AI for a patient-centered approach, teaching AI to those within the healthcare systems to ensure local applicability, providing technology and AI education in LMICs, and multidisciplinary education involving computer science students to address the ethical and technical aspects of AI in medicine.

User:

•8.Authentic assessment in medical education: exploring AI integration and student-as-partners collaboration.pdf

Kimi:

First Author’s Country: Pakistan

Article Language: English

Article Publication Date: Accepted 3 July 2024

Article Type: On Reflection (Commentary)

Applications of Artificial Intelligence/Large Language Models in Medical Education/Training mentioned in the article: 1. Generative AI for creating new learning materials, personalized practice problems, and simulations. 2. S2AG tools for delivering concise, tailored responses from existing knowledge sources. 3. Immersive AR/VR simulations for realistic, interactive learning environments. 4. ChatGPT’s performance on the United States Medical Licensing Exam and its potential in medical education. 5. AI integration in simulated workplace-based assessment (SWBA) for surgical education. 6. AI tools for creating authentic assessments, such as SimInsights, AccuTouch, MedCognition, Simucase, iHuman, Surgical Mentor, ProMIS, Isabel Healthcare, Infermedica, and PathChat.

Ethical Challenges or Difficulties or Issues mentioned in the article: 1. Over-reliance on technology diminishing critical hands-on skills. 2. Risks of AI-driven assessments, including data privacy, security standards, and ethical guidelines. 3. Ensuring fairness and preventing biases in assessments across diverse student backgrounds and abilities. 4. Maintaining academic integrity and preventing plagiarism or cheating in the context of real-world data use for assessments.

Suggestions or Solutions for these Ethical Challenges: 1. Balanced approaches integrating AI with traditional teaching methods to maintain student engagement and practical skill development. 2. Clear regulatory frameworks to ensure compliance with data privacy, security standards, and ethical guidelines. 3. Policymaker prioritization of resource allocation for equitable access to AI technologies. 4. Quality assurance mechanisms to monitor and enhance the reliability of AI algorithms used for assessments. 5. Faculty development programs in assessment to establish accountability, motivate learning, and create effective assessment practices.

User:

•9.Twelve tips for addressing ethical concerns in the implementation of artificial intelligence in medical education.pdf

Kimi:

First Author’s Country: Australia

Language of the Article: English

Publication Date: 03 April 2024

Type of Article: Article

Applications of AI/Large Language Models in Medical Education/Training:

•Personalized learning experiences

•Enhanced diagnostic training

•Hands-on experience without risk

•Augmented decision-making skills

•Streamlined administrative tasks

•Instant access to a wide range of information

•Technology-enhanced teaching

•Adaptive assessments

•Immediate contextual feedback

•Collaborative learning through virtual platforms

•Support faculty in creating advanced, data-driven curricula

Ethical Challenges/Difficulties/Issues Mentioned:

•Privacy issues related to handling and security of sensitive student and patient data

•Biases inherent in AI algorithms leading to unequal educational opportunities or misinterpretations in clinical training

•Lack of transparency in AI decision-making processes

•Potential devaluation of human judgment as AI becomes more integrated into educational frameworks

•The lack of AI expertise in faculty

•The lack of standard guidelines for the responsible use of AI

Suggestions/Solutions for Ethical Challenges:

1.Emphasizing transparency in AI systems development and deployment

2.Addressing biases in AI algorithms to ensure fair representation

3.Validating the content generated by AI educational tools for accuracy

4.Prioritizing data protection and privacy

5.Obtaining informed consent from students and stakeholders

6.Fostering collaboration between AI experts, educators, and students

7.Training educators on AI ethics

8.Empowering students with knowledge about AI

9.Regularly monitoring AI algorithms for performance and accuracy

10.Establishing accountability for AI system outcomes

11.Adhering to standard guidelines and regulatory awareness

12.Forming an ethics committee to address ethical issues

Note: The document does not contain any charts or figures, so no additional information regarding ethics from charts has been extracted.

User:

•10.Metaverse and its impact on medical education and health care system A narrative review.pdf

Kimi:

First Author’s Country: Iran

Language of the Article: English

Publication Date: 2024

Type of Article: Narrative Review

Applications of AI/Large Language Models in Medical Education/Training:

•The metaverse, combining AI, AR/VR, and other technologies, offers innovative solutions in medical education.

•Virtual platforms enhance medical education and strengthen medical skills and patient communication.

•The metaverse facilitates collaborative learning, simulation-based training, and remote access to medical resources.

•AR/VR technologies are used in anatomy, radiology, and surgical education.

•The metaverse allows for risk-free surgical training and remote surgical assistance.

Ethical Challenges/Difficulties/Issues Mentioned:

•Privacy and security concerns with the use of data generated by the metaverse.

•The potential for the metaverse to be used for dangerous activities due to its freedom and fewer rules compared to the real world.

•Ethical concerns in new medical scenarios due to advancements in medical science and technology.

•The need for clear legal frameworks, especially for medical purposes, to safeguard the privacy and integrity of health information.

Suggestions/Solutions for Ethical Challenges:

•Obtaining approval from medical regulatory systems like HIPAA and PIPEDA before using metaverse technologies for medical purposes.

•Ensuring databases collecting and storing health care system data maintain confidentiality to avoid unauthorized disclosure of patient information.

•Prioritizing patient privacy protection to foster mutual trust, especially in psychiatry where VR/AR technologies are employed.

•Implementing decisive security measures to prevent theft and unauthorized access to patient information, considering the potential weaknesses of XR systems against cyberattacks and hacking.

User:

•11.AI-Powered Chatbots in Medical Education Potential Applications and Implications.pdf

Kimi:

First Author’s Country: United States

Language of the Article: English

Publication Date: 08/10/2023

Type of Article: Review

Applications of AI/Large Language Models in Medical Education/Training:

•Chatbots as interactive tutors and learning tools, providing personalized assistance, real-time feedback, and guiding students through challenging concepts.

•Automation of repetitive tasks such as creating flashcards, developing acronyms and mnemonics, and summarizing complex topics.

•Future use in presenting clinical scenarios for simulated patient presentations and decision-making skills training.

•Assistance in creating board-style questions or simulated clinical assessments.

•Serving as a point-of-care reference, providing clinical information in an understandable and interactive format.

•Assistance in patient education by generating health-related articles and materials.

•Support in keeping up with medical literature by classifying and categorizing research articles.

•Facilitation of the research process by aiding in literature reviews and statistical analysis.

Ethical Challenges/Difficulties/Issues Mentioned:

•Chatbots cannot fulfill the human experience and may have potential biases within the practice of medicine.

•The technology is only as effective as its programming and the data it is trained on, which may lead to inaccurate information if the input data is incomplete or biased.

•Limited access to chatbot technology may perpetuate educational disparities.

•The potential for chatbots to falsify and create references, known as “hallucination.”

•The need for clear citation of sources to ensure students are not misled by inaccurate or incomplete information.

Suggestions/Solutions for Ethical Challenges:

•Establishing frameworks and tools to integrate chatbots into existing medical education programs.

•Developing chatbot platforms specifically for medical education with content aligned with standardized curriculum and learning objectives.

•Ensuring chatbots are designed and implemented ethically and effectively, including establishing guidelines and best practices.

•Training medical educators and students on how to use chatbots effectively.

•Conducting rigorous evaluations of chatbot performance and impact.

•Involving stakeholders in the development and implementation of chatbots in medical education.

User:

•12.Needs, Challenges, and Applications of Artificial Intelligence in Medical Education Curriculum.pdf

Kimi:

First Author’s Country: United States

Language of the Article: English

Publication Date: 07/06/2022

Type of Article: Review

Applications of AI/Large Language Models in Medical Education/Training:

•AI is anticipated to become integral in patient care, physician’s office, and surgical suites, influencing clinical decision-making with AI-driven predictions.

•Medical schools should incorporate AI in the curriculum to educate students about the breadth of AI tools, the framework of engineering and designing AI solutions to clinical issues, and the role of data in AI innovations.

•Study cases in the curriculum should include AI recommendations to present critical decision-making challenges.

•AI should be integrated across different aspects of the curriculum to lower costs, improve quality, and expand access to healthcare.

Ethical Challenges/Difficulties/Issues Mentioned:

•The shift from the clinician-patient dyad to a clinician-AI-patient triad introduces ethical, legal, and financial implications.

•Minority exclusions in databases, issues with legal protections, and a decrease in humanistic touch are among the ethical issues raised by AI implementation in healthcare.

•The need for an ethical approach to incorporating AI in practice and evolving standards of care is emphasized.

Suggestions/Solutions for Ethical Challenges:

•Medical schools should educate students on the ethical implications of AI in medicine, ensuring that future physicians are knowledgeable about AI’s shortfalls such as transparency and liability.

•Students should develop abilities to distinguish correct information from rhetoric and understand how to create and disseminate thoroughly validated, trustworthy information for patients and the public.

•Medical education governing bodies, deans, and researchers should work together to implement AI in the undergraduate medical education curriculum, ensuring ethical considerations are at the forefront.

User:

•13.Leveraging ChatGPT for ophthalmic education A critical appraisal.pdf

Kimi:

First Author’s Country: India

Language of the Article: English

Publication Date: 6 November 2023

Type of Article: Editorial

Applications of AI/Large Language Models in Medical Education/Training:

•ChatGPT can offer real-time, customized learning experiences in ophthalmic education.

•It can simulate patient interactions and clinical scenarios to improve diagnostic and interpersonal skills.

•ChatGPT can be used for supplemental learning, reinforcing knowledge acquired through traditional methods.

•It can facilitate interactive learning experiences, group discussions, and collaborative learning in a teaching environment.

Ethical Challenges/Difficulties/Issues Mentioned:

•Concerns about reliability, lack of personal touch, and potential biases in AI-generated content.

•Ethical considerations concerning data privacy and potential misuse of AI.

•The dynamic nature of ophthalmological diseases requires continuous model training and validation on diverse and updated datasets.

•Obtaining informed consent for using patient data for continuous training is crucial.

•The reliance on AI for diagnostics and treatment decisions might impact the trust and rapport between the patient and the ophthalmologist.

•Addressing the limitations of ChatGPT regarding the lack of recency in rapidly evolving fields like ophthalmology is vital.

Suggestions/Solutions for Ethical Challenges:

•Developers should continue refining ChatGPT to recognize and avoid promoting misinformation.

•Transparent disclosure that ChatGPT is an AI language model should be a priority.

•Establishing regulatory frameworks and guidelines for responsible AI use in ophthalmology, including standardized protocols for model validation, addressing bias, ensuring data security, and establishing accountability in case of erroneous predictions leading to patient harm.

User:

•14.Application of artificial intelligence in medical education focus on the application of ChatGPT for clinical medical education.pdf

Kimi:

First Author’s Country: Republic of Korea

Language of the Article: English

Publication Date: June 2023

Type of Article: Review Article

Applications of AI/Large Language Models in Medical Education/Training:

•AI, specifically ChatGPT-3.5, is used to simulate authentic clinical scenarios for enhancing learners’ diagnostic and communication skills.

•AI is utilized to generate role-play scripts based on clinical ethics cases, which are then reviewed and optimized by medical professors.

•AI technology is integrated into medical education to overcome limitations in developing role-play scripts within conventional educational settings.

Ethical Challenges/Difficulties/Issues Mentioned:

•The AI-generated role-play scripts may not always be flawless and require expert review to ensure alignment with real-world clinical situations.

•There are concerns about the AI’s ability to generate content that matches the educational goals and content accuracy.

•Ethical issues related to data privacy and the potential misuse of AI in medical education are acknowledged.

Suggestions/Solutions for Ethical Challenges:

•Addressing the limitations and ethical concerns requires a combination of AI technology and human expertise, where AI-generated content is reviewed and refined by medical professionals.

•It is suggested that future studies should focus on overcoming these limitations while further investigating the potential applications of AI in medical education.

•Establishing ethical standards and guidelines for the use of AI in medical education to ensure responsible and accountable use of the technology.

User:

•15.Artificial intelligence in medical education - perception among medical students..pdf

Kimi:

First Author’s Country: India

Language of the Article: English

Publication Date: 2024

Type of Article: Research

Applications of AI/Large Language Models in Medical Education/Training:

•AI is viewed as an assistive technology for reducing medical errors.

•AI can enhance medical decision accuracy.

•AI has the potential to improve accessibility to healthcare.

•AI can facilitate patient education and increase patient control over their health.

Ethical Challenges/Difficulties/Issues Mentioned:

•Concerns about AI replacing physicians.

•Fear of a reduction in the humanistic aspect of medicine.

•Challenges to trust in patient-physician relationships due to AI-assisted applications.

•Risks of violations of professional confidentiality.

Suggestions/Solutions for Ethical Challenges:

•Structured training on AI applications during medical education, particularly on ethical issues.

•Understanding AI’s role in healthcare as an assistant rather than a replacement for physicians.

•Addressing misconceptions about AI and fostering a meticulous understanding of its role in healthcare.

•Integrating soft skills, such as compassion and empathy, alongside AI education to ensure future healthcare professionals can navigate the intersection of technology and patient-centered care.

User:

•16.Medical Students’ Attitudes toward AI in Medicine and their Expectations for Medical Education.pdf

Kimi:

First Author’s Country: Germany

Language of the Article: English

Publication Date: November 1, 2023

Type of Article: Preprint

Applications of AI/Large Language Models in Medical Education/Training:

•AI is perceived as reliable, trustworthy, and technically competent in the medical context.

•Medical students are interested in learning more about AI opportunities in medicine.

•Basic AI knowledge, including modes of operation, ethics, areas of application, reliability, and possible risks, should be taught in medical studies.

Ethical Challenges/Difficulties/Issues Mentioned:

•Ethical concerns about AI use in medicine were highlighted by participants.

•Issues of data protection and privacy due to the use of sensitive patient health data in AI applications.

•Transparency and explainability of AI systems, which can make complex and opaque decisions.

•Discrimination and bias in AI systems due to insufficient or biased training data.

Suggestions/Solutions for Ethical Challenges:

•Medical education should address ethical issues by teaching challenges of data protection and privacy.

•It is important to teach the sensible use of human oversight and continuous monitoring to catch errors or biases in AI algorithms.

•Ensure that final decisions are made by human clinicians to maintain the ethical use of AI in medicine.

User:

•17.AI-Enabled Medical Education Threads of Change, Promising Futures, and Risky Realities Across Four Potential Future Worlds..pdf

Kimi:

First Author’s Country: United States

Language of the Article: English

Publication Date: December 25, 2023

Type of Article: Perspective

Applications of AI/Large Language Models in Medical Education/Training:

•AI is anticipated to transform various aspects of medical education, including personalized learning, fair access to resources, and enhancing patient-practitioner communication.

•AI can facilitate personalized medicine, early disease detection, and prevention through efficient data analysis.

•AI can support drug discovery, clinical trials, and public health by aiding in trial design and resource management.

•AI can be used in medical education to provide customized and engaging learning through AI tutors and mentors, offering varied educational content and assessments.

Ethical Challenges/Difficulties/Issues Mentioned:

•Ethical management of AI is crucial to ensure its responsible use and equal benefit distribution.

•Concerns about AI-generated disinformation, loss of privacy, widening inequality, erosion of human autonomy, and ethical dilemmas.

•The potential for AI to standardize education at the expense of critical thinking, creativity, and social skills.

Suggestions/Solutions for Ethical Challenges:

•Develop a robust ethical framework that emphasizes patient safety, privacy, and autonomy while promoting equity and inclusivity.

•Foster interdisciplinary collaboration to encourage cooperation among various experts in developing and implementing AI technologies, ensuring they address complex needs and challenges in healthcare and medical education.

•Invest in education and training to prepare professionals and trainees with necessary skills and knowledge to effectively use and critically evaluate AI technologies.

User:

•18.Anesthesiologists’ Perspective on the Use of Artificial Intelligence in Ultrasound-Guided Regional Anaesthesia in Terms of Medical Ethics and Medical Education A Survey Study..pdf

Kimi:

First Author’s Country: Turkey

Language of the Article: English

Publication Date: June 27, 2023

Type of Article: Original Article

Applications of AI/Large Language Models in Medical Education/Training:

•AI is used to improve medical education, particularly in ultrasound-guided regional anesthesia (RA), by assisting in the identification of sono-anatomy.

•AI can identify vital sono-anatomical structures, display targets, and guide practitioners to the correct targets.

•AI is expected to increase safety, decrease complication rates, and enhance educational aspects of RA.

•AI is seen as beneficial for patient care, resident training, post-graduate medical education, and reducing complications in practice.

Ethical Challenges/Difficulties/Issues Mentioned:

•Concerns about who will be held accountable for inaccuracies in AI-generated content.

•Privacy and data governance issues, particularly regarding the storage of sonographic images in AI programs.

•The potential for AI to replace human decision-making and the ethical implications of AI’s role in medical practice.

Suggestions/Solutions for Ethical Challenges:

•Developers should be clear about data security measures in AI.

•Address practitioners’ concerns and potential users’ ethical concerns through studies.

•Establish guidelines and regulations for AI use in medicine, addressing issues like accountability for errors, data governance, and privacy.

•Encourage interdisciplinary collaboration to address medicolegal and ethical aspects of AI.

User:

•19.Reflections on the Development of Large Language Models and Their Impact on Basic Medical Education.pdf

Kimi:

First Author’s Country: South Korea

Language of the Article: English

Publication Date: June 2024

Type of Article: Review Article

Applications of AI/Large Language Models in Medical Education/Training:

•Personalized learning experiences by adjusting the level of explanation based on the user’s needs.

•Broadening access to a wide range of medical knowledge and streamlining literature reviews to support research.

•Providing dynamic and immersive educational experiences through interactive learning environments that mimic clinical settings.

•Facilitating the simulation of clinical scenarios for diagnosis and management practice.

Ethical Challenges/Difficulties/Issues Mentioned:

•Concerns about the accuracy and reliability of information provided by LLMs.

•Ethical implications of using AI, including potential biases and the need for transparency.

•The risk of over-reliance on technology, which may affect the development of critical thinking skills.

Suggestions/Solutions for Ethical Challenges:

•Adopt LLMs based on evidence-based reviews to ensure the accuracy and reliability of information.

•Foster multidisciplinary collaboration to address the complex needs and challenges in healthcare and medical education.

•Ensure transparency in the use of AI to build trust among patients, healthcare professionals, and students.

•Manage cognitive load effectively to prevent over-reliance on AI and maintain critical thinking skills.

User:

•20.The rise of ChatGPT Exploring its potential in medical education.pdf

Kimi:

First Author’s Country: Republic of Korea

Language of the Article: English

Publication Date: 2023

Type of Article: Viewpoint Commentary

Applications of AI/Large Language Models in Medical Education/Training:

•ChatGPT can serve as a virtual teaching assistant, providing students with detailed and relevant information.

•It can be used to automate time-intensive tasks such as summarizing and evaluating research and medical literature.

•ChatGPT has the potential to offer real-time and personalized feedback to students.

•Integration with other technologies, such as gaming industry tools, can develop interactive virtual laboratory simulations relevant to medical practice.

•ChatGPT can generate self-check quizzes with answer explanations.

Ethical Challenges/Difficulties/Issues Mentioned:

•Ensuring the accuracy and reliability of information provided by AI systems in medical education.

•Concerns about students becoming overly reliant on AI models, potentially impacting the development of critical thinking and problem-solving skills.

•The risk of academic dishonesty or “cheating” facilitated by AI tools.

•Potential for AI models to perpetuate biases and discrimination if trained on biased data or designed with inherent bias.

•Ethical and legal concerns related to privacy and confidentiality in the context of sensitive information sharing in medical education.

•The possibility of AI systems replacing human educators, leading to job loss and a reduction in personal attention and feedback for students.

•Accountability and transparency issues in the use of AI in medical education, particularly in cases of errors or mistakes made by AI systems.

Suggestions/Solutions for Ethical Challenges:

•Develop clear guidelines and verification mechanisms to ensure the accuracy of AI systems within medical education.

•Provide proper training for students on how to use AI tools and evaluate the information they return to prevent over-reliance and academic dishonesty.

•Incorporate assessments that require critical thinking, creativity, and synthesis of information that cannot be easily completed with language models to discourage cheating.

•Use plagiarism detection technologies to counter academic dishonesty.

•Address potential biases in AI models by ensuring diverse and representative training data and by designing algorithms that minimize discrimination.

•Implement measures to protect privacy and confidentiality when using AI tools in medical education, including careful evaluation of potential risks and ensuring adequate safeguards are in place.

•Consider the implications of AI on job markets and develop policies and initiatives to support educators and mitigate adverse effects on employment.

User:

•21.Artificial Intelligence in Undergraduate Medical Education A Scoping Review.pdf

Kimi:

Extracted Information from the Article

1.First Author’s Country: Canada

2.Language of the Article: English

3.Publication Date: The article was published in 2020.

4.Type of Article: Article (specifically, a scoping review)

Applications of Artificial Intelligence in Medical Education/Training

The article discusses several applications of artificial intelligence (AI) in undergraduate medical education (UME):

•Curriculum Development: There is a recognized need for an AI curriculum in UME, which includes machine learning literacy and AI ethics.

•Skills Development: Medical students should learn how to work with and manage AI systems, including understanding foundational statistical concepts, AI fundamentals, and the implications of AI on clinical reasoning.

•Ethics and Legal Implications: The curriculum should also address the ethical and legal implications of AI systems in clinical practice, fostering discussions around liability and data privacy.

•Critical Appraisal: Students should be equipped with skills to critically appraise AI systems and understand their strengths and weaknesses.

Ethical Challenges Mentioned in the Article

The article identifies several ethical challenges related to the integration of AI in medical education:

•Liability Issues: Questions about who is responsible when AI systems make errors.

•Data Privacy: Concerns regarding the handling of patient data and the potential for breaches of confidentiality.

•Bias in AI Systems: The risk of AI systems perpetuating or exacerbating existing biases in healthcare.

Suggestions or Solutions for Ethical Challenges

To address these ethical challenges, the article suggests:

•Frameworks for AI Ethics: Providing students with frameworks to approach ethical issues related to AI, including discussions on liability and data privacy.

•Transparency in AI Development: Emphasizing the importance of developing transparent AI tools that can be understood by healthcare professionals.

•Interdisciplinary Collaboration: Encouraging collaboration between medical educators, ethicists, and data scientists to create a comprehensive understanding of AI ethics.

User:

•22.Impact of Large Language Models on Medical Education and Teaching Adaptations..pdf

Kimi:

Extracted Information from the Article

1.First Author’s Country: China

2.Language of the Article: English

3.Publication Date: July 25, 2024

4.Type of Article: Article (Viewpoint)

Applications of Artificial Intelligence/Large Language Models in Medical Education/Training

The article discusses the following applications of AI/LLMs in medical education:

•Enhancing Teaching Quality: LLMs can improve the efficiency and quality of instruction by generating comprehensive syllabi, engaging lecture materials, and detailed textbooks.

•Personalized Learning: LLMs offer a flexible, digital, and immediate approach to learning, tailoring instructional content to the specific needs, abilities, interests, and learning styles of students.

•Strengthening Clinical Skills Training: LLMs can simulate standardized patients for clinical skills training, addressing limitations such as high costs, temporal and spatial constraints, and patient privacy issues.

•Optimizing Teaching Assessment Processes: LLMs can generate a variety of assessment questions, provide immediate feedback, and offer insights into examination results.

•Boosting the Efficiency of Medical Research: LLMs can assist in information retrieval, summarize lengthy papers, and facilitate data analysis and visualization in medical research.

•Supporting Continuing Medical Education: LLMs can help medical professionals stay up-to-date with the latest developments in medical research and clinical practices.

Ethical Challenges Mentioned in the Article

The article identifies several ethical challenges related to the use of LLMs in medical education:

•Accuracy of Information: Concerns about the generation of fabricated data, incorrect citations, and AI hallucination.

•Overreliance and Academic Integrity: Risk of plagiarism and academic dishonesty due to the use of LLMs for writing papers and preparing for exams.

•Bias and Transparency: Potential biases in the training data and algorithms, as well as the lack of transparency in LLMs’ decision-making processes.

•Privacy and Data Security: Risks of patient privacy breaches and cybersecurity risks associated with data storage and processing.

Suggestions or Solutions for Ethical Challenges

The article suggests the following measures to address these ethical challenges:

•Critical Scrutiny: Educators should verify AI-generated content with references to trusted sources and teach students to discern the truthfulness of information.

•Clear Policies on Academic Misconduct: Educational institutions should establish explicit policies on academic misconduct related to the use of LLMs.

•Promoting Critical Thinking: Educators should foster a critical perspective among students regarding the outputs of LLMs and encourage reflective practices.

•Emphasis on Practical Experience: Emphasize hands-on experience and experiential learning in authentic clinical settings to ensure the development of empathy and clinical skills.

•Data Privacy Protection Education: Provide comprehensive education and training on patient privacy protection, including lawful and ethical procedures for the collection, use, and storage of patient information.

User:

•23.Patient Autonomy in Medical Education Navigating Ethical Challenges in the Age of Artificial Intelligence.pdf

Kimi:

Extracted Information from the Article

1.First Author’s Country: China

2.Language of the Article: English

3.Publication Date: 2024

4.Type of Article: Article

Applications of Artificial Intelligence in Medical Education/Training

The article discusses the following applications of AI in medical education:

•Enhanced Diagnostics and Treatment: AI is used to improve diagnostic accuracy and enable personalized medicine.

•Simulation of Patient Cases: AI is used to simulate patient cases for educational purposes.

•Assistance in Diagnosis and Medical Data Analysis: AI aids in diagnosis and analyzes medical data.

•Educational Tools: AI is integrated into medical education to assist in various educational activities.

Ethical Challenges Mentioned in the Article

The article identifies several ethical challenges related to the use of AI in medical education:

•Data Privacy and Security: Concerns about how AI systems handle, process, and store sensitive personal medical data.

•Bias in Algorithms: AI algorithms may perpetuate biases or inequalities present in the data they are trained on.

•Informed Consent: Ensuring that patients are fully informed and consent to the use of AI in their healthcare.

•Algorithmic Transparency: The need for transparency in AI algorithms used in healthcare decision-making.

Suggestions or Solutions for Ethical Challenges

The article suggests the following measures to address these ethical challenges:

•Interdisciplinary Collaboration: Collaboration with fields such as computer science, data ethics, law, and social sciences to provide a holistic understanding of the ethical landscape.

•Ethical Education in Curricula: Updating medical curricula to incorporate ethics related to AI, fostering interdisciplinary collaboration, and recommending policies to ensure ethical standards in AI-driven healthcare environments.

•Critical Assessment of AI Systems: Equipping students with the knowledge and tools to critically assess AI systems for biases and disparities.

•Respect for Patient Autonomy: Empowering patients to make decisions about their healthcare, including the use of AI.

User:

•24.A systematic review of large language models and their implications in medical education.pdf

Kimi:

Extracted Information from the Article

1.First Author’s Country: China

2.Language of the Article: English

3.Publication Date: 2023

4.Type of Article: Article

Applications of Artificial Intelligence in Medical Education/Training

The article discusses the following applications of AI in medical education:

•Digital Health Education: Preparing the next generation of physicians with digital health knowledge and skills.

•Clinical Decision Support Systems (CDSS): Utilizing AI for Adjuvant therapy/diagnosis/analytics and data ranking.

•Telemedicine: The use of digital technologies in medical practice and research.

•Big Medical Data: The application of large-scale medical data in healthcare.

Ethical Challenges Mentioned in the Article

The article identifies several ethical challenges related to the use of AI in medical education:

•Data Privacy and Security: Concerns about the protection of personal health information in digital health education.

•Ethical Framework: The need for an ethical framework to guide the application of digital technologies in health, especially with regards to AI.

•Legal Knowledge: The importance of understanding legal issues related to digital health.

•Equity in Digital Health Education: Regional imbalances in the availability and quality of digital health education.

Suggestions or Solutions for Ethical Challenges

The article suggests the following measures to address these ethical challenges:

•Inclusion of Ethics in Medical Curriculum: Ethics knowledge must be included in the medical curriculum, especially regarding AI and digital technologies.

•National Initiatives: A call for national initiatives to support the adoption of systematic approaches to curriculum design in digital health.

•Interdisciplinary Collaboration: Encouraging collaboration between medical education, computer science, and finance to provide a broader development space for medical students.

•Students as Partners (SaP) Approach: Engaging students in the co-creation of learning and teaching materials, which can enhance the curriculum development process.

User:

•25.The need for digital health education among next-generation health workers in China a cross-sectional survey on digital health education.pdf

Kimi:

Extracted Information from the Article

1.First Author’s Country: China

2.Language of the Article: English

3.Publication Date: 2023

4.Type of Article: Article

Applications of Artificial Intelligence in Medical Education/Training

The article discusses the following applications of AI in medical education:

•Digital Health Education: Preparing the next generation of physicians with digital health knowledge and skills.

•Clinical Decision Support Systems (CDSS): Utilizing AI for辅助治疗/diagnosis/analysis and data ranking.

•Telemedicine: The use of digital technologies in medical practice and research.

•Big Medical Data: The application of large-scale medical data in healthcare.

Ethical Challenges Mentioned in the Article

The article identifies several ethical challenges related to the use of AI in medical education:

•Data Privacy and Security: Concerns about the protection of personal health information in digital health education.

•Ethical Framework: The need for an ethical framework to guide the application of digital technologies in health, especially with regards to AI.

•Legal Knowledge: The importance of understanding legal issues related to digital health.

•Equity in Digital Health Education: Regional imbalances in the availability and quality of digital health education.

Suggestions or Solutions for Ethical Challenges

The article suggests the following measures to address these ethical challenges:

•Inclusion of Ethics in Medical Curriculum: Ethics knowledge must be included in the medical curriculum, especially regarding AI and digital technologies.

•National Initiatives: A call for national initiatives to support the adoption of systematic approaches to curriculum design in digital health.

•Interdisciplinary Collaboration: Encouraging collaboration between medical education, computer science, and finance to provide a broader development space for medical students.

•Students as Partners (SaP) Approach: Engaging students in the co-creation of learning and teaching materials, which can enhance the curriculum development process.

User:

•26.Ethical use of artificial intelligence in health professions education AMEE Guide No.158..pdf

Kimi:

Extracted Information from the Article

1.First Author’s Country: Oman

2.Language of the Article: English

3.Publication Date: 2023

4.Type of Article: Article (AMEE Guide)

Applications of Artificial Intelligence in Medical Education/Training

The article discusses the following applications of AI in medical education:

•Enhancing Learning Outcomes: Using data-driven decisions to provide the best possible learning outcomes for each student.

•Predicting Behavior Patterns: Predicting student behavior patterns to improve educational strategies.

•Simulation and Training: Utilizing AI for simulating patient cases and assisting in diagnosis, which can revolutionize how healthcare professionals are trained.

•Personalized Education: AI systems can offer personalized education by tracking student progress and adapting content accordingly.

Ethical Challenges Mentioned in the Article

The article identifies several ethical challenges related to the use of AI in medical education:

•Data Privacy and Security: Concerns about the collection, storage, and use of student data, especially with the increase in data gathering capabilities of AI.

•Informed Consent: Ensuring that students are fully informed and consent to the use of their data for AI applications.

•Bias in Algorithms: The potential for AI algorithms to perpetuate biases present in the training data, affecting fairness and impartiality in educational settings.

•Transparency and Explainability: The lack of transparency in AI algorithms, which can hinder understanding and trust in AI systems used in education.

•Responsibility and Accountability: Determining who is responsible for decisions made by AI systems and the outcomes of those decisions.

•Autonomy: The impact of AI on student and teacher autonomy, as AI systems may influence educational choices and paths.

•Beneficence: Ensuring that the use of AI in medical education leads to positive outcomes and does not harm students or compromise their rights.

Suggestions or Solutions for Ethical Challenges

The article suggests the following measures to address these ethical challenges:

•Establish Education Ethics Committees: Set up committees similar to Research Ethics Boards to focus on AI issues in education, especially concerning the use of student data.

•Educate on AI Ethics: Ensure that members of Research Ethics Boards and other relevant committees are educated on AI ethics and its implications for research and education.

•Chief AI Ethics Officer: Appoint a Chief AI Ethics Officer to guide AI policy in the institution, ensuring ethical use of AI in education and research.

•Transparent Algorithms: Use open-source algorithms where possible and ensure that AI systems are well-documented to increase transparency.

•Informed Consent Processes: Strengthen informed consent processes to ensure students understand how their data will be used and have a say in its use.

•Data Ownership and Control: Address the issue of data ownership, ensuring students have control over their data and are acknowledged for their contribution to AI systems.

•Legal and Ethical Compliance: Ensure compliance with data protection laws and adapt to the evolving legal landscape regarding AI and data use.

User:

•27.A checklist for reporting, reading and evaluating Artificial Intelligence Technology Enhanced Learning (AITEL) research in medical education.pdf

Kimi:

Extracted Information from the Article

1.First Author’s Country: Oman

2.Language of the Article: English

3.Publication Date: 16 Jan 2024

4.Type of Article: Article

Applications of Artificial Intelligence in Medical Education/Training

The article discusses the use of Artificial Intelligence Technology Enhanced Learning (AITEL) in medical education, focusing on the need for a systematic approach to reporting, reading, and evaluating research involving AI systems. It emphasizes the importance of understanding the AI systems used in medical education research, which can include various applications such as:

•AI Systems as Interventions: AI systems are increasingly used as interventions in medical education research, where they are applied to enhance learning outcomes.

•Technology Enhanced Learning (TEL): AI is integrated into TEL systems to create AITEL systems, introducing new complexities and risks that require detailed reporting.

Ethical Challenges Mentioned in the Article

The article identifies several ethical challenges related to the use of AI in medical education:

•Data Privacy and Security: Ensuring that training data sets used in AI systems are free of errors and address ethical concerns such as privacy and confidentiality.

•Bias in AI Systems: Reducing or understanding bias in training data to ensure fair and non-discriminatory AI applications in education.

•Legal Compliance: Adhering to international standards and laws related to AI use in education, which may impact future research grant applications and journal requirements.

•Transparency and Accountability: Ensuring transparency in AI algorithms and accountability for the outcomes of AI systems in education.

Suggestions or Solutions for Ethical Challenges

The article suggests the following measures to address these ethical challenges:

•Comprehensive Reporting: Introduces an AITEL Reporting Checklist to guide researchers in reporting on AI systems, covering protocols, modeling, algorithm design, training data, testing, and validation.

•Ethical Considerations in Research Protocols: Researchers should include ethical considerations in their study protocols, addressing issues like data privacy, security, and bias.

•Legal Compliance: Researchers should be aware of and comply with international laws and standards related to AI in education, such as those from the European Commission.

•Transparency in AI Algorithms: Clear identification of algorithms and algorithm types should be provided to reduce the ‘black box’ nature of AI systems.

User:

•28.Ethical risks in robot health education A qualitative study.pdf

Kimi:

Extracted Information from the Article

1.First Author’s Country: China

2.Language of the Article: English

3.Publication Date: 2024

4.Type of Article: Article (Empirical Paper)

Applications of Artificial Intelligence in Medical Education/Training

The article discusses the use of health education robots in medical education, focusing on their potential as a support force in nursing practice. The applications include:

•Health Education Delivery: Using robots to disseminate scientific knowledge, concepts, and skills in the health field to patients and their families.

•Interactive Functions: Engaging in simple human-robot interaction with minimal physical contact, which is perceived to entail low ethical risk.

Ethical Challenges Mentioned in the Article

The article identifies several ethical challenges related to the use of health education robots in medical education:

•Threats to Human Dignity: Concerns about patients feeling devalued or objectified due to interactions with robots.

•Concerns about Patient Safety: Doubts about the robots’ educational capability and potential risks to patient safety.

•Apprehensions about Privacy Disclosure: Anxiety regarding the leakage of personal information and the disclosure of sensitive medical conditions.

•Worries about Implicit Burdens: Increased workload due to the need to maintain and operate robots, and the potential for robots to become tools for clinical assessments, adding pressure on nursing staff.

•Concerns about Responsibility Attribution: Determining accountability in cases of errors or accidents involving robots.

•Expectations for Social Support: The need for government, social, and hospital support to facilitate the integration of robots in healthcare.

Suggestions or Solutions for Ethical Challenges

The article suggests the following measures to address these ethical challenges:

•Adherence to RRI Principles: Ensuring that the design, production, operation, and application of robots are guided by ethical values, including respect for patient dignity and autonomy.

•Data Privacy Protection: Establishing unified standards for data encryption, access control, and identity authentication to safeguard patient information.

•Training for Healthcare Workers: Providing proper training to prevent misuse or operational errors that may lead to liability.

•Legislative and Regulatory Support: Enacting legal regulations to standardize ethical risks and clarify the obligations and responsibilities of stakeholders.

•Public Awareness and Acceptance: Promoting public understanding and acceptance of robots in healthcare through educational campaigns and media outreach.

•Inclusive Stakeholder Engagement: Involving a broad range of stakeholders, including patients, in the development and ethical review process of robotic health education technologies.

User:

•29.The Pros and Cons of Using ChatGPT in Medical Education A Scoping Review..pdf

Kimi:

Extracted Information from the Article

1.First Author’s Country: Qatar

2.Language of the Article: English

3.Publication Date: 2023

4.Type of Article: Scoping Review

Applications of Artificial Intelligence/Large Language Models in Medical Education/Training

The article discusses the following applications of ChatGPT in medical education:

•Automated Scoring: ChatGPT is used for automatic scoring/grading in medical education.

•Teaching Assistance: It aids in academic and scientific writing, research papers, and creating new educational content.

•Personalized Learning: ChatGPT provides quick access to information, enhancing personalized learning.

•Research Assistance: It assists with research tasks and generating case scenarios and exam questions.

•Content Creation: Educators use ChatGPT to create new content for learning facilitation.

•Language Translation: ChatGPT is used for language translation in clinical radiology.

Ethical Challenges Mentioned in the Article

The article identifies several ethical challenges related to the use of ChatGPT in medical education:

•Risk of Incorrect Information: ChatGPT may provide incorrect or inaccurate information.

•Fabricated References: It generates false and misleading references that appear genuine.

•Ethical Issues: Concerns about exam cheating and biased responses to certain queries.

•Transparency Issues: Issues related to the transparency and copyright of content generated by ChatGPT.

•Declining Critical Thinking Abilities: The potential undermining of students’ critical thinking skills.

Suggestions or Solutions for Ethical Challenges

The article suggests the following measures to address these ethical challenges:

•Standardized Evaluation Tools: Developing standardized evaluation tools to assess the benefits and risks of using ChatGPT in medical education.

•Caution in Use: Emphasizing the need to use ChatGPT with caution due to its limitations and challenges.

•Focus on Future Studies: Encouraging future research to examine the use of ChatGPT in medical education.

User:

•30.Artificial Intelligence in Surgical Training for Kidney Cancer A Systematic Review of the Literature.pdf

Kimi:

First Author’s Country: Italy Article Language: English Article Publication Date: 27 September 2023 Article Type: Article

Applications of Artificial Intelligence/Large Language Models in Medical Education/Training mentioned in the article: 1. Surgical skill assessment and performance evaluation. 2. Automated analysis of surgical video for workflow analysis and intraoperative guidance. 3. Objective assessment of actions, errors, and risks during surgery. 4. Identification and estimation of surgical instruments’ pose and movement. 5. Enhancement of surgical simulations and robot-assisted surgery. 6. Integration with virtual reality (VR) and augmented reality (AR) for surgical training. 7. Creation of personalized e-learning platforms and content. 8. Provision of personalized quantitative feedback on technical proficiency. 9. Use of AI-tutoring systems for proficiency-based training and skill improvement.

Ethical challenges or difficulties or problems mentioned in the article: 1. Data privacy. 2. Transparency. 3. Biases. 4. Accountability. 5. Liabilities.

Suggestions or solutions for these ethical challenges: 1. Recognition and resolution of ethical risks associated with AI integration in robotic surgical training. 2. Addressing the need for standardized metrics for objective evaluation of trainees’ surgical skills. 3. Ensuring the development and validation of AI algorithms using diverse datasets to avoid overfitting and improve generalizability. 4. Enhancing the understandability of complex machine learning algorithms to facilitate rigorous testing. 5. Encouraging the creation of verified benchmark datasets in the medical field. 6. Prioritizing scientific problem-solving over publication optimization in research. 7. Adopting sound statistical practices and robust evaluation methods beyond benchmark performance.

User:

•31.Artificial Intelligence Literacy Developing a Multi-institutional Infrastructure for AI Education.pdf

Kimi:

First Author’s Country: United States Article Language: English Article Publication Date: July 2023 Article Type: Article

Applications of Artificial Intelligence/Large Language Models in Medical Education/Training mentioned in the article: 1. AI in radiology literacy course for residents. 2. Remote learning format for AI education. 3. Hands-on clinical AI session with an FDA-cleared AI-assisted viewer and reporting system. 4. Lectures covering basic AI terms and methods, clinical applications of AI in radiology by different subspecialties, and special topics including the economics of AI, ethics of AI, algorithm bias, and medicolegal implications of AI in medicine.

Ethical challenges or difficulties or problems mentioned in the article: 1. Ensuring AI advances health equity instead of deepening health disparities. 2. Role of physicians and researchers in ethical AI implementation. 3. Data security of patient’s Protected Health Information (PHI).

Suggestions or solutions for these ethical challenges: 1. Including a discussion on ethics of AI in the curriculum to highlight the role of physicians and researchers in ensuring AI promotes health equity. 2. Addressing the need for maintaining the role of radiologists as the final interpreters in the ethical implementation of AI. 3. Ensuring data security measures are in place to protect patient’s PHI when using AI algorithms.

User:

•32.Perceptions of Faculty and Students About Use of Artificial Intelligence in Medical Education A Qualitative Study..pdf

Kimi:

First Author’s Country: Saudi Arabia Article Language: English Article Publication Date: April 04, 2024 Article Type: Article

Applications of Artificial Intelligence/Large Language Models in Medical Education/Training mentioned in the article: 1. AI as a source of literature and an extensive information pool for research. 2. Standardization of evaluation. 3. Support in active learning, MCQs construction, scenario generation, training organization, and case simulation. 4. AI applications in patient triage, radiology, and pathology. 5. Use of AI in daily academic activities such as constructing exam questions, research ideas, brainstorming, language and grammar correction, finding answers and information, summarization, looking for resources for research, and doing assignments.

Ethical challenges or difficulties or problems mentioned in the article: 1. Threats to academic integrity. 2. Plagiarism and not using own thoughts. 3. Privacy and confidentiality issues. 4. Lack of respect for intellectual property. 5. Questionable interpretation of information. 6. AI systems not being culture-sensitive and mainly based on Western values and cultures. 7. Misuse of AI as a tool.

Suggestions or solutions for these ethical challenges: 1. Reviewing AI-generated work for accuracy and validity. 2. Ensuring proper citation and attribution to maintain academic integrity. 3. Addressing privacy and confidentiality concerns by implementing strict data protection measures. 4. Respecting intellectual property rights and avoiding plagiarism. 5. Developing AI systems that are culturally sensitive and adaptable to different cultural contexts. 6. Regulating the use of AI in medical education to preserve ethics and responsibility.

User:

•33.Developing Medical Education Curriculum Reform Strategies to Address the Impact of Generative AI Qualitative Study..pdf

Kimi:

First Author’s Country: Japan Article Language: English Article Publication Date: 2023 Article Type: Original Paper

Applications of Artificial Intelligence/Large Language Models in Medical Education/Training mentioned in the article: 1. Assistance in creating instructional content and materials. 2. Improvement of teaching and learning efficiency. 3. Easier generation of virtual cases for clinical education. 4. Support for students in summarizing information and gathering information. 5. Use in adaptive and personalized learning environments. 6. Generation of educational materials by teachers. 7. Support for group learning and case-based learning. 8. Use of digital patients for clinical case-based learning.

Ethical challenges or difficulties or problems mentioned in the article: 1. Concerns about the authenticity of information provided by GAI. 2. Ethical issues related to the use of GAI. 3. Copyright and portrait rights issues. 4. Decreased ability of learners to think independently. 5. Inhibition of existing assessment methods. 6. Superficial academic learning in cognitive areas.

Suggestions or solutions for these ethical challenges: 1. Understanding pitfalls of GAI and learning how to use GAI in the clinical and learning process. 2. Implementing information ethics education to address ethical considerations. 3. Ensuring proper use of GAI in medicine, including ethics and compliance. 4. Maintaining GAI-free learning processes to foster higher cognitive domains of learning. 5. Promoting interactive learning and improving humanistic professional skills, such as empathy.

User:

•34.Artificial Intelligence in Medical Education and Mentoring in Rehabilitation Medicine..pdf

Kimi:

First Author’s Country: United States Article Language: English Article Publication Date: November 2024 Article Type: Original Paper

Applications of Artificial Intelligence/Large Language Models in Medical Education/Training mentioned in the article: 1. Development of new or improved teaching strategies. 2. Personalized education plans. 3. Interactive learning experiences. 4. AI-assisted training systems to enhance technical performance scores. 5. Virtual mentoring platforms to host a wide range of AI mentors. 6. AI platforms to generate data-driven insights from educational interactions. 7. AI-based conversational agents to provide emotional support and motivation.

Ethical challenges or difficulties or problems mentioned in the article: 1. Academic integrity (e.g., appropriate use of AI by students completing assignments). 2. Bias affecting students or patients (e.g., in student evaluations or access to care). 3. Data protection and its impact on patient care. 4. User liability in clinical care.

Suggestions or solutions for these ethical challenges: 1. Teaching AI ethics in medical education to address issues like informed consent, bias, safety, transparency, patient privacy, and allocation. 2. Continuous evaluation and improvement of AI-enabled educational programs. 3. Collaborative efforts between academia, industry, and policymakers to advance research and responsible implementation of AI in medical education.

User:

•35.ChatGPT as a Tool for Medical Education and Clinical Decision-Making on the Wards Case Study..pdf

Kimi:

First Author’s Country: United States Article Language: English Article Publication Date: 2024 Article Type: Original Paper

Applications of Artificial Intelligence/Large Language Models in Medical Education/Training mentioned in the article: 1. Addressing medical knowledge gaps through discrete medical knowledge inquiries. 2. Building differential diagnoses and engaging dual-process thinking. 3. Challenging medical axioms. 4. Using cognitive aids to support acute care decision-making. 5. Facilitating conversations with subspecialties. 6. Engaging in difficult conversations with patients. 7. Exploring ethical challenges and general medical ethics teaching. 8. Personal continuing medical education resources. 9. Developing ward-based teaching tools. 10. Supporting and automating clinical documentation. 11. Supporting productivity and task management.

Ethical challenges or difficulties or problems mentioned in the article: 1. LLM biases. 2. Misinformation. 3. Ethics. 4. Health equity. 5. Data privacy and security. 6. Potential nefarious uses.

Suggestions or solutions for these ethical challenges: 1. Develop a code of conduct for the use of LLMs in clinical and training settings. 2. Independently verify and validate any answers produced by LLMs and not make medical decisions based on LLM outputs unless their accuracy is confirmed. 3. Be honest with patients and colleagues when using LLMs in clinical work. 4. Commit to placing patient care, safety, and trust above any educational or research use of the technology. 5. Abide by the institution’s information technology policies and practices.

User:

•36.Artificial intelligence for healthcare and medical education a systematic review..pdf

Kimi:

First Author’s Country: China Article Language: English Article Publication Date: July 30, 2023 Article Type: Review Article

Applications of Artificial Intelligence/Large Language Models in Medical Education/Training mentioned in the article: 1. Clinical specialty training and continuing education. 2. Radiology, diagnostics, surgery, cardiology, and dentistry. 3. Assisting physicians in improving efficiency and accuracy. 4. Virtual reality, 5G, cloud computing technology, big data analysis, wearable devices, the Internet of Things. 5. New medical courses covering computer science, coding, algorithms, and electrical engineering.

Ethical challenges or difficulties or problems mentioned in the article: 1. Privacy breaches and stigma associated with data, such as chronic illnesses. 2. Lack of unified standards and norms in AI technology. 3. Public skepticism about AI due to uneven application of different AI technologies.

Suggestions or solutions for these ethical challenges: 1. Integrate AI with healthcare and develop standards to reach a common understanding and regulate the use of AI. 2. Develop accreditation standards and licenses for AI applications in healthcare. 3. Increase investment and focus on “AI+Healthcare Education”. 4. Encourage the design of a comprehensive artificial intelligence curriculum by the government.

User:

•37.Demystifying AI Current State and Future Role in Medical Education Assessment..pdf

Kimi:

First Author’s Country: United States Article Language: English Article Publication Date: April 2024 Supplement Article Type: Review Article

Applications of Artificial Intelligence/Large Language Models in Medical Education/Training mentioned in the article: 1. Personalizing the educational experience through precision education. 2. Enhancing learning efficiency and patient outcomes. 3. Reducing resource constraints through data-driven approaches. 4. Reducing bias in assessments. 5. Transforming raw data into meaningful, individualized feedback. 6. Fostering a continuous learning journey from medical school to residency and practice. 7. Providing real-time feedback and support in clinical scenarios. 8. Identifying trainees at risk during their transition to graduate medical education (GME) for targeted early interventions. 9. Analyzing electronic medical record (EMR) entries or student notes for quality patient care or medical knowledge. 10. Predicting patient outcomes based on technical skills in procedural specialties.

Ethical challenges or difficulties or problems mentioned in the article: 1. Algorithmic transparency. 2. Data privacy. 3. Potential for bias propagation. 4. Over-reliance on AI. 5. Unexplained disparities in assessment.

Suggestions or solutions for these ethical challenges: 1. Develop systems that leverage AI while ensuring data that are meaningful, attributable, automatable, scalable, and real-time (TRACER framework). 2. Ensure education leaders understand the AI tools they integrate into their programs. 3. Compare output of performance metrics in different populations to detect bias. 4. Develop AI to proactively detect bias in assessment and flag comments that might contain bias. 5. Use AI to reduce unexplained disparities in assessment by selecting different features than human evaluators.

User:

•38.Practical Use of ChatGPT in Psychiatry for Treatment Plan and Psychoeducation..pdf

Kimi:

First Author’s Country: Iran Article Language: English Article Publication Date: 15 November 2023 Article Type: Review Article

Applications of Artificial Intelligence/Large Language Models in Medical Education/Training mentioned in the article: 1. Therapy and counseling techniques. 2. Self-help and coping strategies. 3. Mindfulness and relaxation techniques. 4. Screening and monitoring. 5. Education and information dissemination. 6. Specialized support for various demographics and conditions. 7. Group and family support. 8. Learning and training for medical professionals. 9. Expressive and artistic therapies. 10. Telepsychiatry and online support. 11. Crisis management and prevention.

Ethical challenges or difficulties or problems mentioned in the article: 1. User privacy. 2. Data security. 3. Potential biases in ChatGPT responses. 4. Misuse and manipulation of AI. 5. Hallucinatory or incorrect outputs from AI.

Suggestions or solutions for these ethical challenges: 1. Establish secure protocols for handling user data. 2. Implement measures to safeguard sensitive information. 3. Actively minimize biases and ensure fair, inclusive, and culturally sensitive responses. 4. Regularly monitor, continuously train, and use diverse training data to mitigate biases. 5. Maintain human oversight and involvement in the therapeutic process. 6. Collaborate between AI developers, mental health professionals, and regulatory bodies to establish ethical guidelines and best practices.

User:

•39.Responsible AI practice and AI education are central to AI implementation a rapid review for all medical imaging professionals in Europe.pdf

Kimi:

First Author’s Country: United Kingdom

Article Language: English

Article Publication Date: July 30, 2023

Article Type: Review Article

Applications of Artificial Intelligence/Large Language Models in Medical Education/Training mentioned in the article:

1.Enhancing medical education through responsible and ethical AI practices.

2.Providing customized training and education for medical imaging professionals.

3.Supporting multidisciplinary collaboration within the AI ecosystem.

4.Improving clinical practice through AI-enabled applications in medical imaging.

5.Offering educational initiatives for radiologists and radiographers to understand AI principles and applications.

6.Facilitating transparency of AI tools through education.

Ethical challenges or difficulties or problems mentioned in the article:

1.Algorithmic transparency and the need for clear understanding of AI decision-making processes.

2.Data privacy concerns regarding the use of patient data in AI applications.

3.Potential biases in AI algorithms that could lead to inequitable healthcare outcomes.

4.The challenge of ensuring that AI tools do not compromise patient safety or quality of care.

5.The need for informed consent from patients regarding the use of their data in AI systems.

Suggestions or solutions for these ethical challenges:

1.Establishing robust governance frameworks for AI tools to ensure ethical and responsible use.

2.Implementing comprehensive training programs for healthcare professionals on AI ethics and applications.

3.Promoting collaboration among stakeholders, including patients, to enhance the quality of AI integration in healthcare.

4.Developing clear guidelines for data privacy and security to protect patient information.

5.Regularly auditing AI systems to identify and mitigate biases, ensuring fair and equitable healthcare delivery.

User:

•40.Performance and exploration of ChatGPT in medical examination, records and education in Chinese Pave the way for medical AI..pdf

Kimi:

First Author’s Country: China Article Language: English Article Publication Date: 2023 Article Type: Article

Applications of Artificial Intelligence/Large Language Models in Medical Education/Training mentioned in the article: 1. Evaluation of ChatGPT’s ability to understand medical knowledge in Chinese. 2. Potential to serve as an electronic health infrastructure for medical development. 3. Performance in medical examinations, records, and education. 4. Assistance in clinical specialty training and continuing education. 5. Support in problem-based learning (PBL) and group discussions.

Ethical challenges or difficulties or problems mentioned in the article: 1. Risks associated with factual errors in NLP, known as “hallucinations.” 2. Economic losses, threats to life and health, legal risks, and ethical issues due to incorrect information. 3. Decline in the overall quality of generated content, leading to a trust hurdle for humans to embrace AI benefits. 4. Concerns about transparency, accountability, and the protection of private data in AI systems.

Suggestions or solutions for these ethical challenges: 1. Develop fundamental principles and requirements for AI use in healthcare. 2. Eliminate hallucinations in large language models to address the trust hurdle. 3. Implement model fine-tuning, develop accountability mechanisms, strengthen human validation and investment. 4. Engage in broad academic debate and focus on quality and transparency in AI development. 5. Prioritize the establishment of trust in AI systems, address transparency concerns, pursue interpretable solutions, and attend to ethical and legal issues related to data protection, commercialization, and ensuring accountability.

User:

•41.Potential and limitations of ChatGPT and generative artificial intelligence in medical safety education..pdf

Kimi:

First Author’s Country of Affiliation: China

Language of the Article: English

Publication Date of the Article: November 16, 2023

Type of Article: Letter to the Editor

Applications of AI/Large Language Models in Medical Education/Training Mentioned in the Article:

•Providing the public with essential knowledge about medications and fostering a scientific approach to drug usage.

•Offering a wealth of knowledge, anonymity, continuous availability, and personalized services.

•Conducting medical safety education with the potential to provide high-quality answers to relevant questions.

•Minimizing user concerns with stigmatization, encouraging honest disclosure of crucial illness-related information.

•Overcoming time, space, and language limitations to provide convenient and efficient pharmaceutical and healthcare services.

•Generating tailored treatment recommendations and personalized medical and health care services based on specific patient data.

Ethical Challenges/Difficulties/Issues Mentioned in the Article:

•Concerns about the accuracy of information provided by AI, which could have severe negative impacts on public health.

•Unclear legal responsibilities and ethical obligations for virtual robots like ChatGPT in the medical field.

•Questions about how to reflect the humanistic care of medicine in interactions with AI and how to protect patients’ personal information.

•The potential for inappropriate use of ChatGPT to pose health risks, such as prolonged screen time or internet addiction.

Suggestions/Solutions for These Ethical Challenges:

•Further integrate ChatGPT with real-life scenarios, using electronic aids for real-time interactions to enhance the accuracy and relevance of medical safety education.

•Establish usage guidelines, industry standards, and regulatory frameworks to regulate the application of ChatGPT in the medical field.

•Provide a clear delineation of safety and responsibility risks to address ethical and safety concerns effectively.

User:

•42.Teaching AI Ethics in Medical Education A Scoping Review of Current Literature and Practices..pdf

Kimi:

First Author’s Country of Affiliation: Austria

Language of the Article: English

Publication Date of the Article: October 16, 2023

Type of Article: Review

Applications of AI/Large Language Models in Medical Education/Training Mentioned in the Article:

•AI is expected to significantly impact patient care, medical research, and the healthcare system.

•AI, particularly machine learning, is used in various medical fields such as radiology, pathology, and dermatology.

•Large language models (LLM), which are part of AI-based chat applications like ChatGPT, are expected to have a significant impact on medical practice.

Ethical Challenges/Difficulties/Issues Mentioned in the Article:

•Patient autonomy, bias, transparency, and potential loss of empathy in the doctor-patient relationship.

•Issues related to data privacy, user liability, and the potential for bias due to unrepresentative training data.

•Concerns about the amplification of biases in data on which statistical AI algorithms are trained.

Suggestions/Solutions for These Ethical Challenges:

•Teaching AI ethics as part of medical curricula to prepare future medical practitioners for ethical challenges posed by AI.

•Emphasizing the importance of ethical principles and standards in the development and use of AI technologies within medical practice.

•Recommending case-based teaching using real-world examples in interactive seminars and small groups to address ethical challenges.

•The need for more empirical studies and foundational definitions of AI ethics to guide the development of teaching content and modalities.

User:

•43.Proposing a Principle-Based Approach for Teaching AI Ethics in Medical Education..pdf

Kimi:

First Author’s Country of Affiliation: Austria

Language of the Article: English

Publication Date of the Article: January 5, 2024

Type of Article: Original Paper

Applications of AI/Large Language Models in Medical Education/Training Mentioned in the Article:

•AI is used in diverse medical specializations, including dermatology, radiology, and pathology.

•AI-based chat applications like ChatGPT are capable of completing the written portion of the United States Medical Licensing Examination and have potential use in providing information on cancer, assisting in clinical diagnoses, authoring scientific research articles, and patient communication.

Ethical Challenges/Difficulties/Issues Mentioned in the Article:

•The use of AI in medicine presents new ethical challenges, such as the potential for discrimination due to biases in the data used for training or effects on patient autonomy.

•There is a near-complete absence of scientific publications on specific teaching content or methods related to AI ethics as part of medical higher education.

Suggestions/Solutions for These Ethical Challenges:

•There is a widespread consensus on the need for AI and AI ethics instruction in medical education, although the current offerings were deemed inadequate.

•All proposed topics for AI ethics education were rated as highly relevant by the medical students surveyed.

•The study emphasizes the importance of integrating the teaching of AI and AI ethics into medical curricula to adequately prepare future medical professionals.

User:

•44.Artificial Intelligence in Medicine Cross-Sectional Study Among Medical Students on Application, Education, and Ethical Aspects..pdf

Kimi:

First Author’s Country of Affiliation: Austria

Language of the Article: English

Publication Date of the Article: February 9, 2024

Type of Article: Viewpoint

Applications of AI/Large Language Models in Medical Education/Training Mentioned in the Article:

•AI has the potential to improve medical care through more accurate diagnosis.

•AI can reduce the burden on the healthcare system by reducing costs and workload.

•AI-based chat applications like ChatGPT can be used for simulated patient-physician interactions, providing a low-risk environment for medical students to practice diagnostic skills and ethical decision-making.

Ethical Challenges/Difficulties/Issues Mentioned in the Article:

•Risk of bias and potential discrimination against patients due to unrepresentative data used to train AI algorithms.

•Misdiagnoses potentially leading to over- or underdiagnosis with substantial consequences for patients.

•Concerns about job displacement due to the use of AI.

•Ethical challenges related to data privacy and the potential for expropriation of personal health data.

Suggestions/Solutions for These Ethical Challenges:

•Integrate AI ethics into medical education to prepare for its potential impacts and challenges.

•Develop a principle-based approach to AI ethics education, building on the 4 principles of medical ethics—autonomy, beneficence, nonmaleficence, and justice—and extending them by integrating 3 public health ethics principles—efficiency, common good orientation, and proportionality.

•Ensure the representation of data used for AI development to avoid bias and discrimination.

•Address potential limitations and errors in AI products to maintain the validity of AI outputs.

•Adapt the process of obtaining informed consent to meet the challenges posed by AI in medicine.

User:

•45.The Intersection of ChatGPT, Clinical Medicine, and Medical Education..pdf

Kimi:

First Author’s Country of Affiliation: Malaysia

Language of the Article: English

Publication Date of the Article: November 21, 2023

Type of Article: Viewpoint

Applications of AI/Large Language Models in Medical Education/Training Mentioned in the Article:

•ChatGPT has the potential to revolutionize medical education by generating human-like responses and covering diverse topics.

•It can be used as a source of medical knowledge,辅助学习。

•Chatbots can provide information to patients, caretakers, and the public,辅助患者参与和教育。

•They can be used for health screening, disease prevention, participant recruitment, data collection, clinical decision support, patient management, drug discovery, and development, and medical writing.

Ethical Challenges/Difficulties/Issues Mentioned in the Article:

•Privacy, data security, and algorithmic transparency and explainability.

•Errors and liability associated with AI medicine.

•The potential for generative language models to generate fabricated and incorrect information, known as “artificial hallucination.”

•Biases in AI models that can lead to unfair and discriminatory results.

•Regulatory issues and the need for a legal framework to address social, ethical, and legal issues associated with AI in healthcare.

•Academic dishonesty and the fulfillment of authorship criteria when using AI in medical writing.

Suggestions/Solutions for These Ethical Challenges:

•Obtain informed consent from patients before ChatGPT accesses their data.

•Ensure that AI applications used in research and clinical medicine are trained on representative and diverse data sets to avoid biases.

•Impose regulatory measures and acts to address social, ethical, and legal issues associated with AI in healthcare.

•Maintain transparency when using ChatGPT in medical writing to avoid academic dishonesty and plagiarism.

•Establish guidelines on the responsible and ethical use of AI models like ChatGPT in medical education to ensure academic integrity, originality, and critical thinking.

User:

•46.Embracing ChatGPT for Medical Education Exploring Its Impact on Doctors and Medical Students.pdf

Kimi:

First Author’s Country of Affiliation: China

Language of the Article: English

Publication Date of the Article: April 10, 2024

Type of Article: Viewpoint

Applications of AI/Large Language Models in Medical Education/Training Mentioned in the Article:

•Enhancing clinical reasoning and decision-making skills.

•Providing personalized and efficient learning experiences.

•Facilitating simulated dialogues, intelligent tutoring, and automated question-answering.

•Supporting problem-based learning (PBL), team-based learning (TBL), case-based learning (CBL), and precision medical education.

•Assisting in medical research by automating literature review, study design proposals, and statistical analysis.

Ethical Challenges/Difficulties/Issues Mentioned in the Article:

•Safeguarding patient data and adhering to data protection regulations.

•Balancing personalized learning with face-to-face interactions to avoid hindering critical thinking and communication skills.

•Addressing the potential devaluation of collaboration in traditional methodologies such as PBL, CBL, and TBL.

•Overreliance on technology, which may hinder critical thinking and hands-on learning.

•Ensuring cultural diversity and inclusivity in medical education.

Suggestions/Solutions for These Ethical Challenges:

•Transparent communication with students, physicians, and patients to ensure understanding of the technology’s purpose and implications, as well as potential risks and benefits.

•Maintaining a balance between personalized learning and face-to-face interactions.

•Integrating ChatGPT with problem-based learning, team-based learning, and case-based learning methodologies to enhance medical education.

•Proper regulation and supervision to ensure a well-rounded learning environment.

•Emphasizing ethical considerations and human-centric approaches in medical education to fully harness the potential of ChatGPT.

User:

•47.Current Status of ChatGPT Use in Medical Education Potentials, Challenges, and Strategies..pdf

Kimi:

First Author’s Country of Affiliation: China

Language of the Article: English

Publication Date of the Article: August 28, 2024

Type of Article: Viewpoint

Applications of AI/Large Language Models in Medical Education/Training Mentioned in the Article:

•Personalized learning support for medical students.

•Simulating clinical scenarios for educational purposes.

•Facilitating teaching and learning processes.

•Curriculum development.

•Clinical support in standardizing procedures, aiding in disease diagnosis, and delivering health education.

•Interdisciplinary collaboration to promote technical advancement and medical treatment efficiency.

Ethical Challenges/Difficulties/Issues Mentioned in the Article:

•Overreliance on ChatGPT leading to a decrease in critical thinking skills.

•Academic plagiarism and the violation of academic integrity.

•Insufficient factualness, timeliness, and interpretability of content generated by ChatGPT.

•Ethical issues including algorithmic discrimination, allocation of medical responsibility, privacy, and security.

•Undermining communication and trust in clinical settings due to the lack of emotional intelligence in AI.

Suggestions/Solutions for These Ethical Challenges:

•Mitigate the risk of overreliance and academic plagiarism through ideological education, fostering comprehensive competencies, and implementing diverse evaluation criteria.

•Optimize ChatGPT’s training data professionally and enhance the transparency of the generation process to ensure content aligns with medical practice standards.

•Address ethical concerns by enhancing value alignment and establishing pertinent legislation or codes of practice, including dealing with algorithmic discrimination, medical responsibility, privacy, and security.

•Maintain the irreplaceability of medical personnel and educators, emphasizing humanistic care and emotional support in healthcare.

User:

•48.Application of artificial intelligence in medical education A review of benefits, challenges, and solutions.pdf

Kimi:

First Author’s Country of Affiliation: Iran

Language of the Article: English

Publication Date of the Article: 2024

Type of Article: Review

Applications of AI/Large Language Models in Medical Education/Training Mentioned in the Article:

•Improved curriculum design and evaluation.

•Implementation of innovative educational methods in clinical settings.

•Use of virtual reality (VR) in fields such as anatomy and surgery.

•Formative and summative assessment processes with less time and cost.

•Clinical setting simulators for stress-free educational environments.

•Increased transparency in education and meaningful comparison of effectiveness across countries and institutions.

•Distance learning and accessibility in areas with limited resources and remote locations.

•Facilitation of active learning strategies such as problem-based, case-based, small-group, and large-group learning.

•Continuous learning opportunities for 24 hours using asynchronous learning methods.

Ethical Challenges/Difficulties/Issues Mentioned in the Article:

•Ethical challenges in clinical and case-based learning involving patient information in databases, such as cyberattacks, patient privacy, confidentiality, and visual security violations.

•Concerns regarding increased cheating and plagiarism with the adoption of AI in medical education.

Suggestions/Solutions for These Ethical Challenges:

•Students need to be educated on the ethical concerns surrounding the application of AI in medicine, including data confidentiality, patient privacy, security, and accountability.

•Educational programs should cover these ethical issues, allowing students to discuss potential solutions and choose the most suitable ones.

•The importance of interdisciplinary collaborations to address and resolve the major obstacles faced by AI in medicine and medical education through constructive discussions and dialogs.

User:

•49.ChatGPT, a Friend or a Foe in Medical Education A Review of Strengths, Challenges, and Opportunities.pdf

Kimi:

First Author’s Country of Affiliation: Iran

Language of the Article: English

Publication Date of the Article: June 2024

Type of Article: Review Article

Applications of AI/Large Language Models in Medical Education/Training Mentioned in the Article:

•Enhanced quality of interaction between medical students and patients.

•Improved education quality.

•Enhanced research opportunities.

•Personalized learning.

•Virtual patient simulations.

•Cost-effectiveness in medical education.

Ethical Challenges/Difficulties/Issues Mentioned in the Article:

•Ethical concerns and privacy issues, especially with the use of personal data in case presentations and problem-based learning (PBL).

•The potential for bias in ChatGPT results or discrimination based on the training data used.

•Transparency issues, particularly with the “black box” algorithm where users cannot observe the method of result presentation.

Suggestions/Solutions for These Ethical Challenges:

•Addressing potential bias, plagiarism, data security, privacy protection, and the risk of discrimination based on input data by creating ethical guidelines specifically for the use of ChatGPT in medical education.

•Emphasizing the teaching of evidence-based medicine (EBM) principles, logical thinking, and clinical reasoning to enable students to effectively deal with major crises and assess the validity of AI results.

•Developing and revising educational curricula to integrate AI models into teaching strategies, create new evaluation methods that emphasize reasoning and logical thinking, update educational practices, and establish educational environments compatible with AI technology.

User:

•50.Ethical Considerations and Fundamental Principles of Large Language Models in Medical Education Viewpoint..pdf

Kimi:

First Author’s Country: Iran

Article Language: English

Publication Date: June 2024

Article Type: Review Article

Applications of AI/Large Language Models in Medical Education/Training:

•Enhanced interaction quality between medical students and patients.

•Improved education quality.

•Enhanced research opportunities.

•Personalized learning.

•Virtual patient simulations.

•Cost-effectiveness.

•Assistance in curriculum development.

•Aid in designing clinical scenarios and imparting clinical reasoning and problem-solving skills.

•Enhancing the quality of interaction between medical students and patients from diverse cultures.

•Providing virtual patient simulation for improving problem-solving skills, decision-making abilities, and clinical reasoning without risking actual patients.

Ethical Challenges/Difficulties/Issues Mentioned:

•Ethical and transparency concerns.

•Limited access to reliable databases.

•Restricted information availability after 2021.

•Limited development of students’ critical thinking ability.

•Risk of generating AI hallucinations.

•Potential for bias in ChatGPT results or discrimination based on the training data utilized.

•Privacy issues, especially with the use of personal data of patients and students.

•The risk of cyber attacks.

•Transparency issues, particularly with the Blackbox algorithm.

Suggestions/Solutions for Ethical Challenges:

•Extensive interdisciplinary research is necessary for the utilization of AI in medical education.

•Regular monitoring of ChatGPT use by medical education experts and providing feedback to educational authorities.

•Establishing a set of principles for utilizing ChatGPT in medical education, considering responsibility, transparency, and effectiveness.

•Addressing ethical concerns such as potential bias, plagiarism, data security, safeguarding individuals’ privacy, and the risk of discrimination based on input data.

•Creating ethical guidelines specifically for the use of ChatGPT in medical education.

•Teaching evidence-based medicine principles, logical thinking, and clinical reasoning to encourage the use of AI in medical education.

•Developing and revising educational curricula to integrate AI models into teaching strategies.

English Summary:

The article discusses the applications of AI, particularly large language models like ChatGPT, in medical education. It highlights several uses, including enhancing interactions between students and patients, improving education quality, and providing personalized learning. However, it also outlines ethical challenges such as transparency issues, limited access to databases, and privacy concerns. The authors suggest interdisciplinary research, regular monitoring, and the establishment of ethical guidelines to address these challenges and ensure the effective and responsible use of AI in medical education.
